# SLC38A4 promotes Kupffer cell phagocytosis and suppresses tumor liver metastasis

**DOI:** 10.1038/s12276-026-01703-5

**Published:** 2026-05-01

**Authors:** Jie Li, Yong-Da Liu, Renjie Wang, Xing-Yi Lin, Yi-Qing Zhu, Wan-Peng Lu, Yan-Fang Tang, Xing-Peng Guo, Yu-Han Ai, Ting-Ting Xu, Sen Lin, Mei Huang, Jinghan Wang, Xiao-Ting Zhu, Ji-Hang Yuan

**Affiliations:** 1 Department of Medical Genetics, Shanghai Key Laboratory of Medical Bioprotection, Key Laboratory of Biological Defense, Ministry of Education, Naval Medical University, Shanghai, China; 2 Department of Colorectal Surgery, Fudan University Shanghai Cancer Center, Shanghai, China; 3 Department of Oncology, Shanghai Medical College, Fudan University, Shanghai, China; 4 Institute of Hepatobiliary and Pancreatic Surgery, Department of Hepatobiliary and Pancreatic Surgery, Shanghai East Hospital, School of Medicine, Tongji University, Shanghai, China; 5 Department of Anatomy and Physiology, Shanghai Jiao Tong University School of Medicine, Shanghai, China

**Keywords:** Metastasis, Cancer microenvironment, Immunosurveillance, Kupffer cells

## Abstract

Metastasis is the main cause of cancer-related death. The liver is an organ with high metastatic tropism for various malignancies. Hepatic-resident macrophage Kupffer cells are the major non-parenchymal cells in the liver that phagocytize disseminated tumor cells to restrict liver metastasis. However, the critical molecules that modulate phagocytosis of tumor cells by Kupffer cells are still largely unknown. Here, we identified SLC38A4 expressed on tumor cells as a critical suppressor during tumor liver metastasis. SLC38A4 enhances the phagocytosis of various tumor cells by Kupffer cells, leading to a reduction in liver metastasis. Mechanistically, SLC38A4 downregulates the expression of “don’t eat me” molecule CD24, which mediates the roles of SLC38A4 in Kupffer cells phagocytosis and tumor liver metastasis. MYC directly binds to *CD24* promoter and activates *CD24* transcription. Through downregulating MYC, SLC38A4 suppresses the expression of CD24. Patients with low SLC38A4 expression in tumors have more liver metastases. This study demonstrates that SLC38A4 promotes Kupffer cell phagocytosis and restricts tumor liver metastasis by suppressing CD24. The SLC38A4/MYC/CD24 axis represents a novel phagocytosis checkpoint for Kupffer cells and a potential therapeutic target for liver metastasis.

## SLC38A4 enhances Kupffer cell phagocytosis to combat liver metastasis

Liver metastasis, a major cause of cancer-related deaths, involves complex interactions between tumor cells and liver components, including Kupffer cells. This study uncovers the role of SLC38A4, a molecule that suppresses liver metastasis by enhancing Kupffer cell phagocytosis of tumor cells. Using gain-of-function and loss-of-function assays, the authors demonstrate that SLC38A4 downregulates the “don’t eat me” signal CD24, promoting tumor cell clearance by Kupffer cells. The study utilized in vivo and in vitro models, including CRISPR–Cas9 screening, to show that SLC38A4 overexpression reduces liver metastases in various cancer types. The findings highlight the SLC38A4/MYC/CD24 axis as a potential therapeutic target for liver metastasis, suggesting that future research could explore this pathway to develop new cancer treatments. This work advances our understanding of liver metastasis and the immune role of Kupffer cells.

This summary was initially drafted using artificial intelligence, then revised and fact-checked by the author.

## Introduction

Metastasis is responsible for ~90% of cancer-related deaths^1^. The liver is a common metastatic target organ for various malignancies, including hepatocellular carcinoma (HCC), colorectal cancer (CRC), pancreatic cancer, neuroendocrine tumor, melanoma, breast cancer, and less commonly, gastrointestinal stromal tumors^2,3^. The liver contains various cell components such as hepatocytes, liver sinusoidal endothelial cells, Kupffer cells, T cells, and natural killer cells^4^. Liver metastasis is influenced by complex interactions between tumor cells and liver cell components^5–8^. However, the critical molecules mediating this interaction and tumor liver metastasis remain largely unknown^9^.

As liver-resident macrophages, Kupffer cells account for ~10% of all liver cells and represent the most abundant portion of liver immune cells^10^. Kupffer cells have critical roles in maintaining the normal physiological state and immune surveillance of the liver^11–17^. A growing body of research highlights the pivotal role of Kupffer cells in defending against liver metastasis by phagocytosing disseminated tumor cells^18–20^. Although certain Kupffer cell-specific molecules, such as ID3 and Dectin-2 (refs. ^18,20^) have been implicated in regulating their phagocytic activity, the tumor-intrinsic factors governing phagocytosis or phagocytosis evasion remain poorly understood^19,21^. In contrast to liver-resident Kupffer cells, the mechanisms by which tumor-intrinsic signals — such as pro-phagocytic “eat me” signals (for example, SLAMF7, calreticulin, and tumor-associated antigens) and anti-phagocytic “don’t eat me” signals (for example, CD47, PD-L1, CD24, and β2-microglobulin) — regulate bone marrow-derived macrophage phagocytosis have been extensively studied^22–28^. CD47 blocking antibodies have been developed to promote the clearance of tumor cells by macrophages^29^. Similarly, further uncovering the tumor-intrinsic mechanisms underlying the evasion of phagocytosis of tumor cells by Kupffer cells holds the potential to yield novel therapeutic strategies for liver metastasis^30^.

In our previous study, in vivo genome-scale CRISPR–Cas9 knockout screening was performed to identify the genes involved in liver metastasis^19^. We noted that the gRNA targeting *Slc38a4* was enriched in liver metastatic tissues. We previously identified the downregulation of SLC38A4 as an oncofetal molecular event in HCC^31^. SLC38A4 was downregulated in fetal liver and HCC, suppressed HCC tumor growth, and its low expression was correlated with poor prognosis in patients with HCC^31^. Another report also showed that low SLC38A4 expression predicted a worse prognosis in CRC^32^. The in vivo genome-scale CRISPR–Cas9 knockout screening data imply that SLC38A4 not only participates in HCC growth but may also be involved in tumor liver metastasis. Here, using gain-of-function and loss-of-function assays, we confirmed that SLC38A4 suppresses liver metastasis of various tumor cells, including HCC, CRC, and melanoma cells. Enhanced phagocytosis of tumor cells by Kupffer cells induced by SLC38A4 contributes to the suppressive roles of SLC38A4 in liver metastasis. Mechanistic exploration revealed that LSC38A4 suppresses the expression of don’t eat me molecule CD24, which mediates the roles of SLC38A4 in Kupffer cells phagocytosis and tumor liver metastasis. SLC38A4 delivers eat me signal to Kupffer cells through downregulation of CD24, leading to the suppression of tumor liver metastasis.

## Materials and methods

### Cell culture

Mouse melanoma cell line B16F10 (cat. SCSP-5233), mouse CRC cell line MC38 (cat. SCSP-5431), mouse HCC cell line Hepa1-6 (cat. SCSP-512), human CRC cell line SW620 (cat. TCHu101), human HCC cell line SNU-398 (cat. SCSP-5206), human melanoma cell line A375 (cat. TCHu155), and human embryonic kidney cell line 293T (cat. SCSP-502) were acquired from the National Collection of Authenticated Cell Cultures in Shanghai, China. B16F10, Hepa1-6, A375, and 293T cells were grown in Dulbecco’s modified Eagle’s medium (Gibco) containing 10% fetal bovine serum (FBS; Gibco). MC38 and SNU-398 cells were cultured in RPMI 1640 medium (Gibco) supplemented with 10% FBS. SW620 cells were cultivated in L-15 medium (Gibco) supplemented with 10% FBS. All cells were incubated at 37 °C in an environment containing 5% CO₂. Their authenticity was verified through short tandem repeat profiling and they were tested and determined to be free of mycoplasma contamination.

### RNA extraction and quantitative PCR (qPCR)

Total RNA was extracted using TRIzol reagent (Invitrogen and Thermo Fisher Scientific). Subsequently, the isolated RNA was reverse-transcribed with HiScript III RT SuperMix for qPCR kit (cat. R323, Vazyme, Nanjing, China). qPCR was performed on a QuantStudio Real-Time PCR Instrument (Applied Biosystems and Thermo Fisher Scientific) using the ChamQ Universal SYBR qPCR Master Mix (cat. Q711, Vazyme). The primer sequences used in the experiment are presented in Supplementary Table 1. β-Actin was used as an endogenous control. Relative expression levels were determined by applying the 2^−ΔΔCt^ method.

### Expression vectors construction, short hairpin RNAs (shRNAs) synthesis, lentiviruses production, and stable cell lines constructions

To construct a vector expressing mouse SLC38A4, mouse *Slc38a4* coding sequences (CDS) were amplified by PCR and subcloned into the BamHI and EcoRI sites of pcDNA3.1/V5-His B Vector (Invitrogen) using NovoRec® plus One step PCR Cloning Kit (Novoprotein, Suzhou, China). To generate mouse SLC38A4 expression lentivirus, mouse *Slc38a4* CDS was subcloned into the NotI and NsiI sites of the lentiviral expression vector LV17 (EF-1a/Luciferase17&Puro) (GenePharma) using the ClonExpress Entry One Step Cloning Kit (Vazyme). The constructed LV17 vector was co-transfected with pGag/Pol, pRev, and pVSV-G into 293T cells using Lipofectamine 3000 (Invitrogen). Lentivirus was harvested 72 h after transfection and then filtered through 0.45 μm polyvinylidene fluoride filters. Human SLC38A4 expression lentivirus was generated, as described previously^31^. The CDS of mouse *Myc* and human *MYC* was PCR-amplified and subcloned into the EcoRI and XbaI sites of the pCMV-N-Flag Vector (Beyotime, Shanghai, China) using the NovoRec plus One step PCR Cloning Kit (Novoprotein) to construct mouse or human MYC expression vectors. The CDS of *Cd24a* was PCR-amplified and subcloned into the BamHI and EcoRI sites of pcDNA3.1/V5-His B Vector (Invitrogen) using NovoRec plus One step PCR Cloning Kit (Novoprotein, Suzhou, China). The primer sequences used for PCR are presented in Supplementary Table 1.

Two independent cDNA oligonucleotides suppressing mouse SLC38A4 expression were designed, synthesized, and subcloned into the lentiviral shRNA vector LV16 (U6/Luciferase17&Puro) (GenePharma, Shanghai, China), designated as mouse sh-SLC38A4-1 and sh-SLC38A4-2. cDNA oligonucleotides suppressing mouse CD24 expression were designed, synthesized, and subcloned into the lentiviral shRNA vector LV3 (H1/GFP&Puro) (GenePharma), designated as mouse sh-CD24. Scrambled non-targeting cDNA oligonucleotides were used as negative control (sh-NC). To generate mouse SLC38A4 or CD24 knockdown lentivirus, sh-SLC38A4-1, sh-SLC38A4-2, or sh-CD24 was co-transfected with pGag/Pol, pRev, and pVSV-G into 293T cells using Lipofectamine 3000 (Invitrogen). Lentivirus was harvested 72 h after transfection and then filtered through 0.45 μm polyvinylidene fluoride filters. The human SLC38A4 knockdown lentivirus was generated, as described previously^31^. Two independent cDNA oligonucleotides suppressing human or mouse MYC expression were designed, synthesized, and subcloned into the SuperSilencing shRNA expression vector pGPU6/Neo (GenePharma, Shanghai, China), designated as sh-MYC-1 and sh-MYC-2. The cDNA oligonucleotide sequences of mouse sh-SLC38A4-1, sh-SLC38A4-2, sh-CD24, human or mouse sh-MYC-1, sh-MYC-2, and sh-NC are listed in Supplementary Table 1.

To generate cells stably overexpressing mouse SLC38A4, Hepa1-6, B16F10, and MC38 cells were infected with mouse SLC38A4 expression lentivirus in the presence of 8 μg/ml polybrene (Sigma-Aldrich). To establish cells stably overexpressing human SLC38A4, SW620 and SNU-398 cells were infected with human SLC38A4 expression lentivirus using 8 μg/ml polybrene (Sigma-Aldrich). To obtain cells with stable knockdown of mouse SLC38A4, Hepa1-6 cells were infected with mouse sh-SLC38A4-1, sh-SLC38A4-2, and sh-NC lentiviruses in the presence of 8 μg/ml polybrene (Sigma-Aldrich). Similarly, for the generation of cells with human SLC38A4 knockdown, SW620 cells and A375 cells were infected with human sh-SLC38A4-1, sh-SLC38A4-2, and sh-NC lentiviruses in the presence of 8 μg/ml polybrene (Sigma-Aldrich). Subsequently, these infected cells were selected with 2 μg/ml puromycin for 4 weeks.

To obtain cells with stable overexpression of mouse or human MYC, mouse or human MYC expression vectors were transfected into Hepa1-6 or SW620 cells, respectively, using Lipofectamine 3000 (Invitrogen), followed by being selected with 200 µg/ml neomycin for 4 weeks. To obtain cells with stable knockdown of mouse or human MYC, sh-MYCs were transfected into Hepa1-6, B16F10, MC38, SW620, SNU-398, and A375 cells using Lipofectamine 3000 followed by being selected with 200 µg/ml (for Hepa1-6, MC38, SW620, SNU-398, and A375) or 800 µg/ml (for B16F10) neomycin for 4 weeks.

To obtain cells with concurrent stable knockdown of SLC38A4 and CD24, Hepa1-6 cells with SLC38A4 knockdown were infected with sh-CD24 lentivirus in the presence of 8 μg/ml polybrene (Sigma-Aldrich), followed by being sorted by fluorescence-activated cell sorting (FACS) using BD FACS Aria II. To obtain cells with concurrent stable overexpression of SLC38A4 and MYC, MYC expression vectors were transfected into B16F10 and SW620 cells with SLC38A4 stable overexpression using Lipofectamine 3000 (Invitrogen), followed by being selected with 2 μg/ml puromycin and 800 µg/ml (for B16F10) or 200 µg/ml (for SW620) neomycin for 4 weeks. To obtain cells with concurrent stable knockdown of SLC38A4 and MYC, sh-MYCs were transfected into Hepa1-6 and A375 cells with SLC38A4 stable knockdown using Lipofectamine 3000, followed by being selected with 2 μg/ml puromycin and 200 µg/ml neomycin for 4 weeks.

### Western blot

Total protein was harvested from the indicated cells using RIPA buffer (Beyotime) supplemented with a protease inhibitor cocktail (Calbiochem, Billerica, MA, USA). The cell lysates were then centrifuged at 12,000 rpm for 5 min at 4 °C. Equal amounts of protein were separated by SDS–PAGE and transferred onto nitrocellulose filter membranes. The membranes were blocked with 5% non-fat dry milk in PBS-T (Tween-20 at a concentration of 0.5 per thousand in PBS) at room temperature for 1 h. Then, they were incubated overnight at 4 °C in PBS-T with primary antibodies against mouse SLC38A4 (20857-1-AP, 1:500, Proteintech, Chicago, IN, USA), human SLC38A4 (ab58785, 1:1,000, Abcam, Cambridge, UK), or β-actin (66009-1-Ig, 1:10,000, Proteintech). After three washes, the membranes were further incubated with IRDye^®^ 680RD goat antimouse IgG secondary antibody (926-68070, 1:10,000, Li-Cor Biosciences, Lincoln, NE, USA) or IRDye 800CW goat anti-rabbit IgG secondary antibody (926-32211, 1:10,000, Li-Cor). The protein bands were detected using an Odyssey infrared scanner (Li-Cor Biosciences). β-Actin was used as a loading control for total protein.

### Animal studies

Male 6–8-week-old athymic BALB/c nude mice and C57BL/6 mice were obtained from the Shanghai Experimental Animal Center of the Chinese Academy of Sciences (Shanghai, China). All mice were maintained in a pathogen-free environment. To detect liver metastasis in vivo, indicated cells were intrasplenically injected into mice. At the specified time, the mice were sacrificed, and their livers were resected, fixed, and subjected to hematoxylin–eosin (HE) staining. The number or area of liver metastases was counted by researchers who were unaware of the experimental group allocation. Mouse experiments were carried out in accordance with animal welfare laws, guidelines, and policies and were approved by the Committee on Ethics of Medicine, Naval Medical University (Shanghai, China).

### Kupffer cells isolation and in vitro phagocytosis analysis

Kupffer cells were isolated as described previously^19^. Briefly, liver tissues were perfused and then dissociated into single-cell suspensions. Hepatocytes were separated from single-cell suspensions by centrifugation at 40×*g* for 3 min. Subsequently, the supernatant was centrifuged at 650×*g* for 7 min to obtain non-parenchymal cells (NPCs). Finally, F4/80^+^ Kupffer cells were isolated from NPCs by magnetic-activated cell isolation (cat. 130-110-443, Miltenyi Biotech).

A total of 0.25 × 10^5^ Kupffer cells co-cultured with 1 × 10^5^ indicated tumor cells labeled with carboxyfluorescein succinimidyl ester (CFSE) using the CellTrace CFSE Cell Proliferation Kit (Thermo Fisher Scientific). The co-culture was carried out in ultra-low-attachment 96-well U-bottom plates (Corning) containing serum-free Iscove’s modified Dulbecco’s medium (Gibco). The cells were incubated at 37 °C in a humidified atmosphere containing 5% CO₂ for 2 h. Following co-culture, cells were collected and stained with an F4/80 antibody (cat. 17-4801-82, eBioscience). Flow cytometry was used to analyze the CFSE intensity within the F4/80^+^ cells. Phagocytosis efficiency was calculated as the ratio of Kupffer cells engulfed CFSE^+^ tumor cells. In CD24 neutralization experiments, 10 μg/ml blocking antibody against human CD24 (cat. 311101, BioLegend) or isotype control (mouse IgG2α, κ; cat. 400201, BioLegend) was added to SW620 cells 30 min before and throughout phagocytosis. Additionally, co-cultured Kupffer cells were pretreated with a mouse Fc-receptor blocking solution.

### In vivo phagocytosis analysis

In vivo phagocytosis assays were performed as described previously^19^. Briefly, the indicated tumor cells, labeled with CFSE, were injected intrasplenically into the indicated mice. Twelve hours later, following perfusion, the livers of mice were dissociated into single-cell suspensions. Hepatocytes were removed from single-cell suspensions by centrifugation at 40×*g* for 3 min. NPCs were obtained by centrifugation of the supernatant at 650×*g* for 7 min. Flow cytometric staining was performed for viable NPCs (negative for 7-AAD; cat. 559925, BD Biosciences or negative for Zombie R718 Fixable Viability Dye; cat. 423115, BioLegend) to detect phagocytosis of CFSE-positive tumor cells by Kupffer cells (characterized as CD45^+^CD11b^int^F4/80^+^). This was achieved using antibodies against CD45.2 (cat. 109807, BioLegend), CD11b (cat. 101215, BioLegend), and F4/80 (cat. 123115, BioLegend).

### Flow cytometry

Flow cytometry analysis was carried out using the Attune NxT Flow Cytometer in conjunction with Attune NxT Software version 5.2 (Thermo Fisher Scientific). The samples were detected using antibodies against human CD24 (cat. 311105, BioLegend), mouse CD24 (cat. 138503, BioLegend), mouse PD-L1 (cat. 124307, BioLegend), human PD-L1 (cat. 329705, BioLegend), mouse CD47 (cat. 127513, BioLegend), or human CD47 (cat. 323123, BioLegend). Data obtained from flow cytometry were processed and analyzed using FlowJo version 10.8.0. To ensure objectivity of the results, the investigators were blinded to the sample identities during flow cytometry.

### Luciferase reporter assay

The promoter regions of mouse *Cd24a* (−800 bp to +100 bp) and human *CD24* (−1,242 bp to −241 bp) were cloned into the XhoI and HindIII sites of the pGL3 basic vector (cat. 212936, Addgene), which is designed to express firefly luciferase. The resulting constructs were designated as pGL3-*Cd24a* promoter and pGL3-*CD24* promoter, respectively. A mouse *Cd24a* promoter mutant was constructed by deleting the sequence from −321 bp to −312 bp, and a human *CD24* promoter mutant was constructed by deleting the sequence from −1,173 bp to −1,164 bp. The primer sequences used in the experiment are presented in Supplementary Table 1.

The pGL3 vectors, SLC38A4 or MYC expression vector, and pRL-TK vector (Promega, Madison, WI, USA) expressing Renilla luciferase were co-transfected into 293T cells using Lipofectamine 3000 (Invitrogen). The pGL3 and pRL-TK vectors were co-transfected into the indicated tumor cells using Lipofectamine 3000 (Invitrogen). Forty-eight hours after transfection, luciferase activity was measured using the Dual-Luciferase^®^ Reporter Assay System (Promega), following the manufacturer’s protocol. The relative firefly luciferase activity was normalized to Renilla luciferase activity.

### Cleavage under targets and release using nuclease (CUT&RUN) assay

CUT&RUN assays were performed using the indicated cells. The assay used the Hyperactive pG-MNase CUT&RUN Assay Kit for PCR/qPCR (cat. HD101, Vazyme) and primary antibodies targeting MYC (cat. 13987, Cell Signaling Technology, Danvers, MA, USA). Enrichment of human *CD24* and mouse *Cd24a* promoters was determined using qPCR. The primer sequences used are presented in Supplementary Table 1.

### Tissue samples

Human HCC, primary CRC, and CRC liver metastatic tissues were collected from patients at Changhai Hospital and Eastern Hepatobiliary Surgery Hospital (Shanghai, China) with informed consent. This study was approved by the Committee on Ethics of Medicine, Naval Medical University (no. NMU-20231008).

### Immunohistochemistry assay

Immunohistochemistry (IHC) staining was performed as described previously with a primary antibody against SLC38A4 (ab58785, 1:40, Abcam), CD24 (10600-1-AP, Proteintech, 1:600) for human clinical samples, CD24 (ab199140, 1:100, Abcam) for mice liver metastatic tissues, or MYC (ab32072, 1:50, Abcam)^33^. Slides were photographed using a Zeiss Axiophot photomicroscope (Carl Zeiss, Oberkochen, Germany).

### Statistical analysis

All statistical analyses were performed using GraphPad Prism software (version 10.0). For comparisons, as specified in the figure legends, Student’s *t* test, one-way analysis of variance followed by Dunnett’s multiple comparisons test, Mann–Whitney test, Kruskal–Wallis test followed by Dunn’s multiple comparisons test, two-way analysis of variance followed by Tukey’s multiple comparisons test, and Spearman correlation analysis were conducted. Statistical significance was defined as *P* < 0.05.

## Results

### SLC38A4 suppresses liver metastasis of various tumor cells

**Fig. 1:**
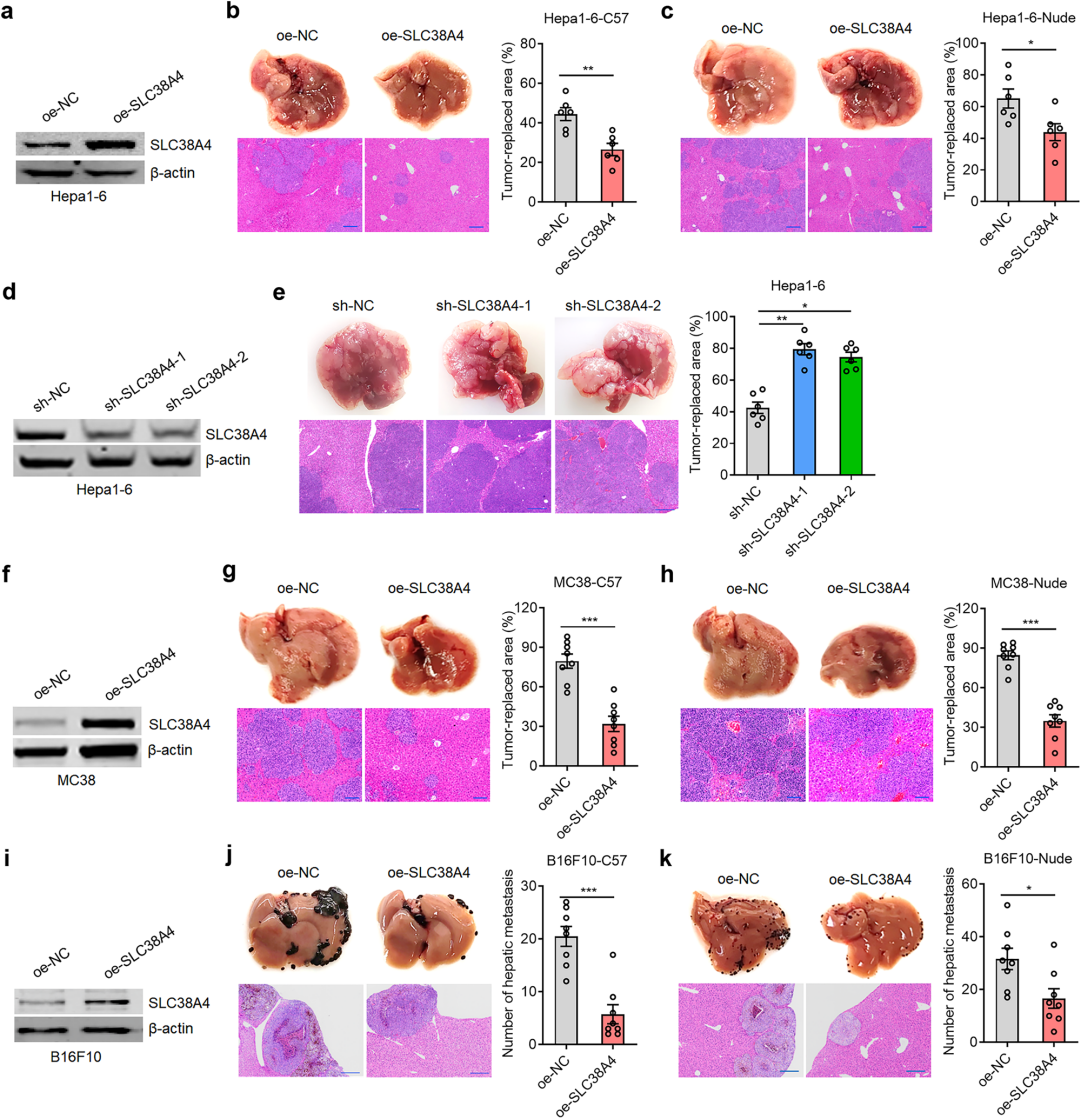
SLC38A4 suppresses tumor liver metastasis. **a** SLC38A4 protein levels in Hepa1-6 cells with SLC38A4 stable overexpression or control were measured by western blot. **b** Representative liver images, hematoxylin–eosin (HE)-stained images of liver tissues isolated from C57BL/6 mice at the 14th day after intrasplenic injection with 1 × 10^7^ indicated Hepa1-6 cells, and tumor-replaced areas are shown. Scale bar, 200 µm. **c** Representative liver images, HE-stained images of liver tissues isolated from nude mice at the 14th day after intrasplenic injection with 1 × 10^7^ indicated Hepa1-6 cells, and tumor-replaced areas are shown. Scale bar, 200 µm. **d** SLC38A4 protein levels in Hepa1-6 cells with SLC38A4 stable knockdown or control were measured by western blot. **e** Representative liver images, HE-stained images of liver tissues isolated from C57BL/6 mice at the 14th day after intrasplenic injection with 1 × 10^7^ indicated Hepa1-6 cells, and tumor-replaced areas are shown. Scale bar, 200 µm. **f** SLC38A4 protein levels in MC38 cells with SLC38A4 stable overexpression or control were measured by western blot. **g** Representative liver images, HE-stained images of liver tissues isolated from C57BL/6 mice at the 8th day after intrasplenic injection with 1 × 10^6^ indicated MC38 cells, and tumor-replaced areas are shown. Scale bar, 100 µm. **h** Representative liver images, HE-stained images of liver tissues isolated from nude mice at the 8th day after intrasplenic injection with 1 × 10^6^ indicated MC38 cells, and tumor-replaced areas are shown. Scale bar, 100 µm. **i** SLC38A4 protein levels in B16F10 cells with SLC38A4 stable overexpression or control were measured by western blot. **j** Representative liver images, HE-stained images of liver tissues isolated from C57BL/6 mice at the 13th day after intrasplenic injection with 1 × 10^6^ indicated B16F10 cells, and hepatic metastasis numbers are shown. Scale bar, 200 µm. **k** Representative liver images, HE-stained images of liver tissues isolated from nude mice at the 9th day after intrasplenic injection with 1 × 10^6^ indicated B16F10 cells, and hepatic metastasis numbers are shown. Scale bar, 200 µm. Results are shown as mean ± s.d. of *n* = 6 (parts **b**, **c** and **e**) or *n* = 8 (parts **g**, **h**, **j** and **k**) mice in each group. *P < 0.05, **P < 0.01, ***P < 0.001 by Mann–Whitney test (parts **b**, **c**, **g**, **h**, **j** and **k**) or Kruskal–Wallis test followed by Dunn’s multiple comparisons test (part **e**). MC mouse colon, NC negative control, SLC solute carrier.

To evaluate the role of SLC38A4 in liver metastasis, we stably overexpressed SLC38A4 in mouse HCC Hepa1-6 cells (Fig. 1a). Intrasplenic injection of tumor cells was performed to establish liver metastasis model. SLC38A4-overexpressing Hepa1-6 cells formed fewer liver metastases in immunocompetent C57BL/6 mice (Fig. 1b), and in *Foxn1*^nu^ nude mice, which lack T cell immunity (Fig. 1c). We further knocked down SLC38A4 in Hepa1-6 cells (Fig. 1d). SLC38A4-deficient Hepa1-6 cells generated more liver metastases in C57BL/6 mice (Fig. 1e). To evaluate whether the pro-metastatic roles of SLC38A4 is HCC-specific, we further overexpressed SLC38A4 in mouse CRC MC38 cells (Fig. 1f). SLC38A4-overexpressing MC38 cells also formed fewer liver metastases in both C57BL/6 and nude mice (Fig. 1g,h). Similarly, SLC38A4 overexpression in mouse melanoma B16F10 cells also suppressed liver metastasis in both C57BL/6 and nude mice (Fig. 1i–k). We further assessed the role of SLC38A4 in human tumor cells. SLC38A4-overexpressing human HCC SNU-398 cells formed fewer liver metastases in nude mice (Supplementary Fig. 1a,b). Likewise, SLC38A4-overexpressing human CRC SW620 cells also formed fewer liver metastases in nude mice (Supplementary Fig. 1c,d). Conversely, SLC38A4-knockdown SW620 cells formed more liver metastases in nude mice (Supplementary Fig. 1e,f). Human melanoma A375 cells with SLC38A4 knockdown similarly exhibited increased liver metastases in nude mice (Supplementary Fig. 1g,h). Together, these results demonstrate that SLC38A4 suppresses liver metastasis of various tumor types.

### SLC38A4 suppresses liver metastasis in a Kupffer cell-dependent manner

We further investigated the mechanism by which SLC38A4 suppresses liver metastasis. Bioluminescence imaging indicated that SLC38A4-overexpressing cells exhibited significantly reduced liver metastasis as early as 12 h post-intrasplenic inoculation (Supplementary Fig. 2a). At this time point, most tumor cells remained in the liver sinusoids, where Kupffer cells actively engulf disseminated tumor cells. To determine whether SLC38A4’s anti-metastatic effect is dependent on Kupffer cells, we depleted Kupffer cells by intraperitoneal injection of clodronate liposomes into C57BL/6 mice. Kupffer cells depletion markedly enhanced tumor liver metastasis (Supplementary Fig. 2b,c). Furthermore, SLC38A4’s liver metastasis-suppressive effect was largely abolished in Kupffer cell-depleted mice (Supplementary Fig. 2b,c). These findings demonstrate that Kupffer cells are the critical mediators of the roles of SLC38A4 in suppressing liver metastasis.

### SLC38A4 enhances phagocytosis of various tumor cells by Kupffer cells

**Fig. 2:**
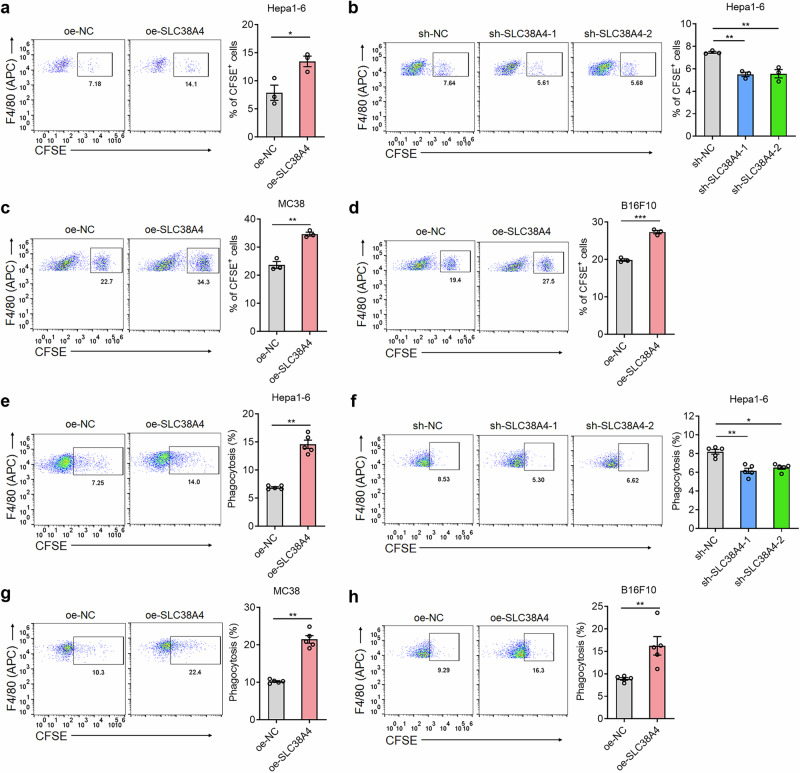
SLC38A4 promotes phagocytosis of tumor cells by Kupffer cells. **a** In vitro phagocytosis of CFSE-labeled Hepa1-6 cells with SLC38A4 overexpression or control by Kupffer cells was measured by flow cytometry. **b** In vitro phagocytosis of CFSE-labeled Hepa1-6 cells with SLC38A4 knockdown or control by Kupffer cells was measured by flow cytometry. **c** In vitro phagocytosis of CFSE-labeled MC38 cells with SLC38A4 overexpression or control by Kupffer cells was measured by flow cytometry. **d** In vitro phagocytosis of CFSE-labeled B16F10 cells with SLC38A4 overexpression or control by Kupffer cells was measured by flow cytometry. **e** In vivo phagocytosis of CFSE-labeled Hepa1-6 cells with SLC38A4 overexpression or control by Kupffer cells was assessed by flow cytometry 12 h later after intrasplenic inoculation. **f** In vivo phagocytosis of CFSE-labeled Hepa1-6 cells with SLC38A4 knockdown or control by Kupffer cells was assessed by flow cytometry 12 h later after intrasplenic inoculation. **g**, In vivo phagocytosis of CFSE-labeled MC38 cells with SLC38A4 overexpression or control by Kupffer cells was assessed by flow cytometry 12 h later after intrasplenic inoculation. **h** In vivo phagocytosis of CFSE-labeled B16F10 cells with SLC38A4 overexpression or control by Kupffer cells was assessed by flow cytometry 12 h later after intrasplenic inoculation. Results are shown as mean ± s.d. of *n* = 3 independent experiments (parts **a**–**d**) or *n* = 5 mice in each group (parts **e**–**h**). *P < 0.05, **P < 0.01, ***P < 0.001 by Student’s *t* test (parts **a**, **c**, and **d**) one-way analysis of variance followed by Dunnett’s multiple comparisons test (part **b**), Mann–Whitney test (parts **e**, **g** and **h**) or Kruskal–Wallis test followed by Dunn’s multiple comparisons test (part **f**). APC allophycocyanin, CFSE carboxyfluorescein succinimidyl ester, MC mouse colon, NC negative control, SLC solute carrier.

Previously, studies have documented that Kupffer cells defending against liver metastasis mainly through phagocytosing disseminated tumor cells^18,34^. Thus, we further investigated whether SLC38A4 modulates phagocytosis of tumor cells by Kupffer cells. CFSE-labeled tumor cells were in vitro co-cultured with Kupffer cells for 2 h, and phagocytosis was quantified by flow cytometry based on CFSE intensity in F4/80^+^ Kupffer cells. The results showed that SLC38A4-overexpressing Hepa1-6 cells showed increased phagocytosis by Kupffer cells (Fig. 2a), whereas SLC38A4-knockdown Hepa1-6 cells showed reduced phagocytosis by Kupffer cells (Fig. 2b). SLC38A4 overexpression in mouse MC38 and B16F10 cells also significantly enhanced phagocytosis of these cells by Kupffer cells (Fig. 2c,d). We further evaluated the effects of SLC38A4 on the phagocytosis of human tumor cells by Kupffer cells. SLC38A4 overexpression in human tumor cells enhanced phagocytosis of these cells by Kupffer cells (Supplementary Fig. 3a,b), whereas SLC38A4 knockdown in human tumor cells decreased phagocytosis of these cells by Kupffer cells (Supplementary Fig. 3c,d).

We next examined the effects of SLC38A4 on Kupffer cell phagocytosis in vivo. Twelve hours after intrasplenic injection of CFSE-labeled tumor cells, in vivo phagocytosis was detected using flow cytometry. The results revealed that SLC38A4-overexpressing Hepa1-6 cells were phagocytosed more efficiently by Kupffer cells in vivo than control Hepa1-6 cells (Fig. 2e), whereas SLC38A4-knockdown Hepa1-6 cells showed reduced phagocytosis by Kupffer cells in vivo (Fig. 2f). SLC38A4 overexpression in mouse MC38 and B16F10 cells also significantly enhanced phagocytosis of these tumor cells by Kupffer cells in vivo (Fig. 2g,h). Consistently, SLC38A4 overexpression in human tumor cells enhanced Kupffer cells-mediated phagocytosis in vivo (Supplementary Fig. 3e,f), whereas SLC38A4 knockdown in human tumor cells suppressed Kupffer cells-mediated phagocytosis in vivo (Supplementary Fig. 3g,h). Together, these results demonstrate that SLC38A4 enhances phagocytosis of various tumor cells by Kupffer cells.

### SLC38A4 downregulates the expression of don’t eat me molecule CD24

**Fig. 3:**
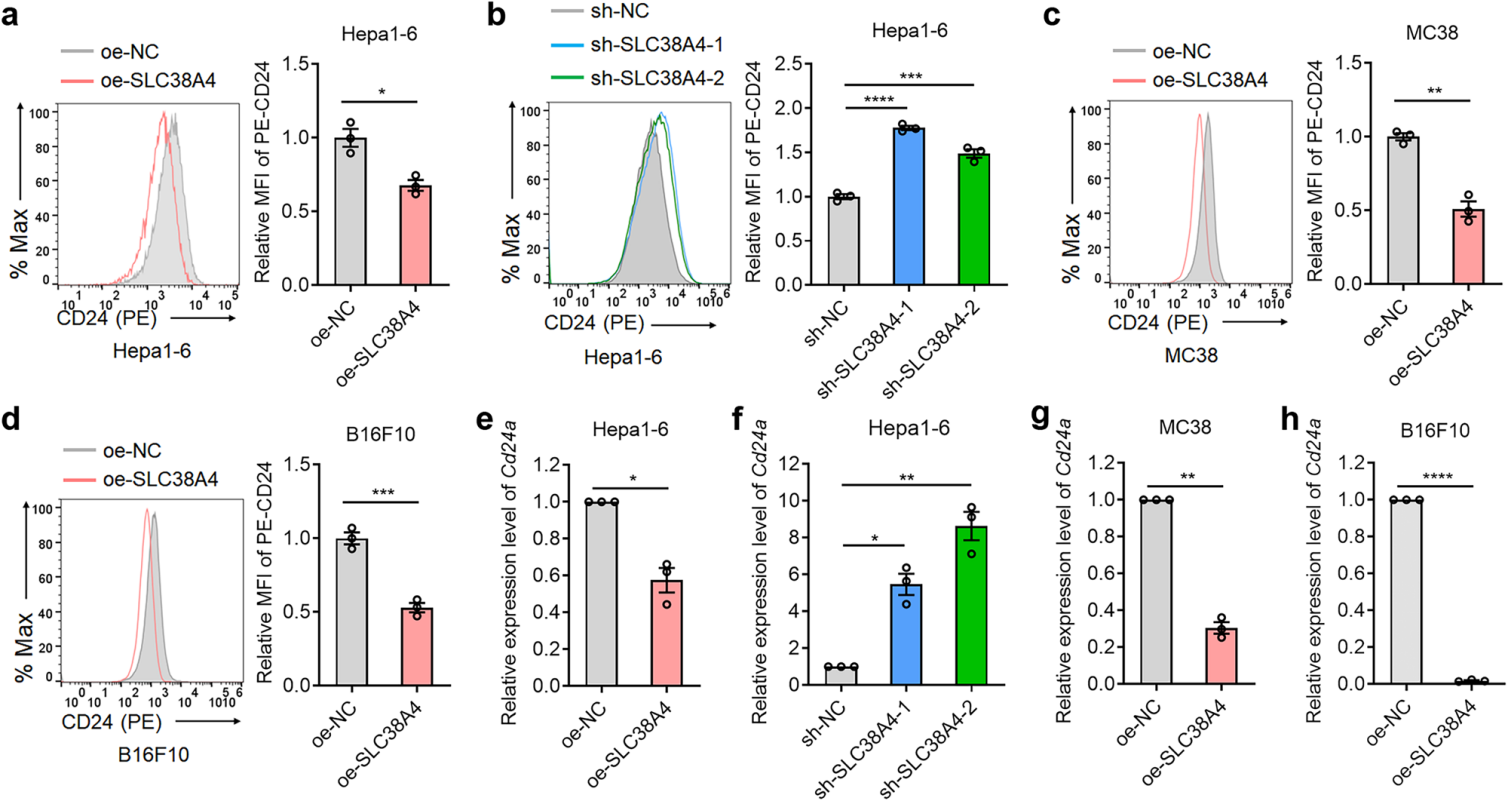
SLC38A4 downregulates CD24 expression. **a** CD24 protein level in Hepa1-6 cells with SLC38A4 overexpression or control was measured by flow cytometry. **b** CD24 protein level in Hepa1-6 cells with SLC38A4 knockdown or control was measured by flow cytometry. **c** CD24 protein level in MC38 cells with SLC38A4 overexpression or control was measured by flow cytometry. **d** CD24 protein level in B16F10 cells with SLC38A4 overexpression or control was measured by flow cytometry. **e** *Cd24a* mRNA level in Hepa1-6 cells with SLC38A4 overexpression or control was measured by quantitative PCR (qPCR). **f** *Cd24a* mRNA level in Hepa1-6 cells with SLC38A4 knockdown or control was measured by qPCR. **g** *Cd24a* mRNA level in MC38 cells with SLC38A4 overexpression or control was measured by qPCR. **h** *Cd24a* mRNA level in B16F10 cells with SLC38A4 overexpression or control was measured by qPCR. Results are shown as mean ± s.d. of *n* = 3 independent experiments. *P < 0.05, **P < 0.01, ***P < 0.001, ****P < 0.0001 by Student’s *t* test (parts **a**, **c**–**e**, **g** and **h**) or one-way analysis of variance followed by Dunnett’s multiple comparisons test (parts **b** and **f**). MC mouse colon, MFI mean fluorescence intensity, NC negative control, PE phycoerythrin, SLC solute carrier.

We next investigated the mechanism by which SLC38A4 enhances phagocytosis of tumor cells by Kupffer cells. We first detected the potential influences of SLC38A4 on the expression of classical don’t eat me molecules CD47, CD24 and PD-L1. Flow cytometry showed that SLC38A4-overexpressing Hepa1-6 cells showed reduced CD24 protein level (Fig. 3a), whereas SLC38A4-knockdown Hepa1-6 cells showed increased CD24 protein level (Fig. 3b). SLC38A4 overexpression in MC38 and B16F10 cells also significantly downregulated CD24 protein level (Fig. 3c,d). Neither overexpression nor knockdown of SLC38A4 changed CD47 protein level (Supplementary Fig. 4a–c). Similarly, PD-L1 protein level was not regulated by SLC38A4 overexpression or knockdown (Supplementary Fig. 4d–f). We further examined the mRNA expression levels of *Cd47*, *CD24a*, and *Cd274* (encoding PD-L1) by qPCR. The results showed that SLC38A4-overexpressing Hepa1-6 cells showed reduced *Cd24a* mRNA level (Fig. 3e), whereas SLC38A4-knockdown Hepa1-6 cells showed increased *Cd24a* mRNA level (Fig. 3f). SLC38A4 overexpression in MC38 and B16F10 cells also significantly downregulated *Cd24a* mRNA level (Fig. 3g,h). Neither overexpression nor knockdown of SLC38A4 changed *Cd47* or *Cd274* mRNA level (Supplementary Fig. 4g–j). We further assess the effects of SLC38A4 on CD24 expression in human cells. Flow cytometry showed that SLC38A4 overexpression in human tumor cells decreased CD24 protein level (Supplementary Fig. 5a,b), whereas SLC38A4 knockdown in human tumor cells increased CD24 protein level (Supplementary Fig. 5c,d). qPCR assays showed that SLC38A4 overexpression in human tumor cells decreased *CD24* mRNA levels (Supplementary Fig. 5e,f), whereas SLC38A4 knockdown in human cells increased *CD24* mRNA levels (Supplementary Fig. 5g,h). Collectively, these data suggest that SLC38A4 downregulates *CD24* expression at the transcriptional level.

### SLC38A4 enhances Kupffer cell phagocytosis and suppresses liver metastasis via CD24 downregulation

**Fig. 4:**
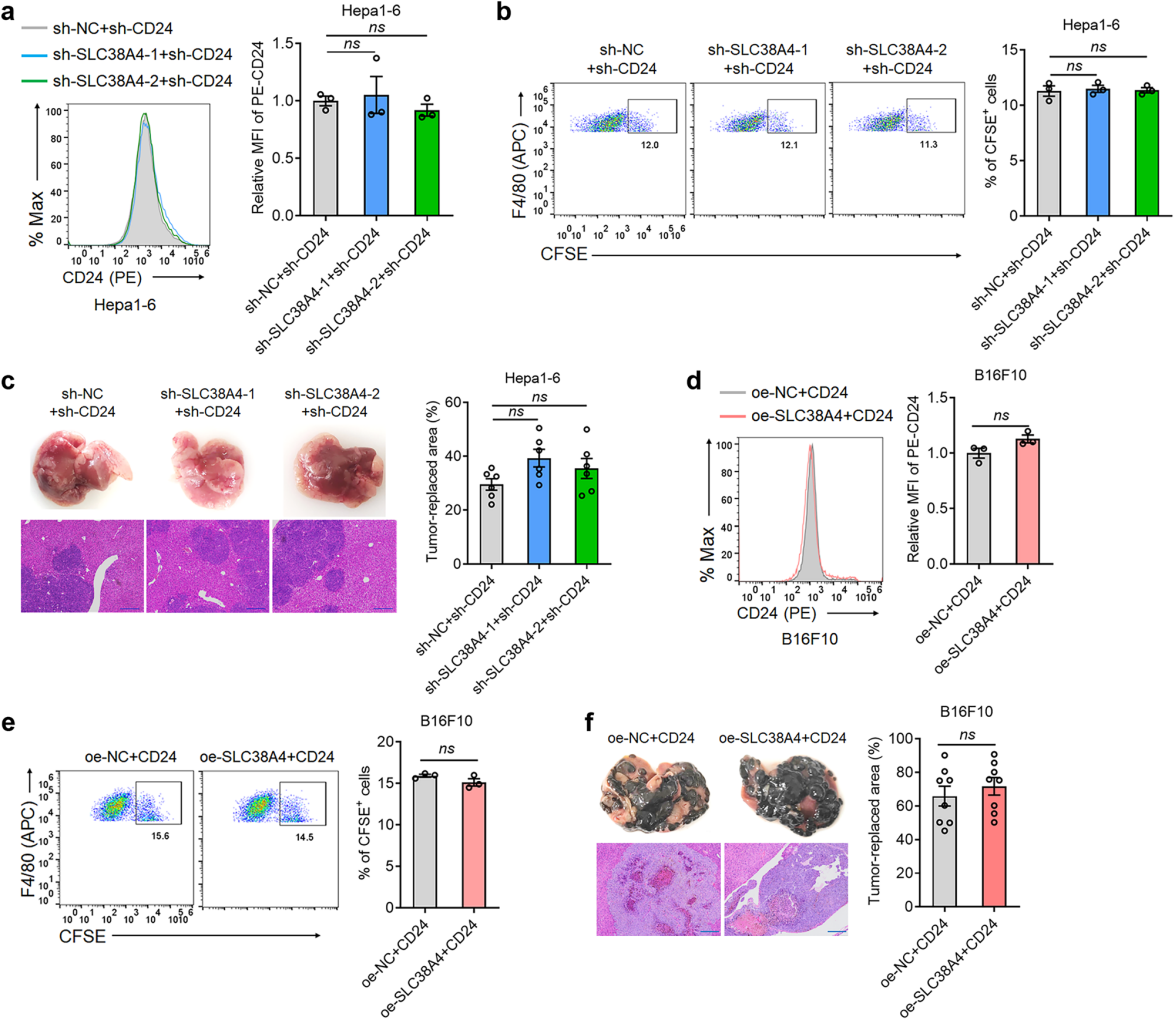
SLC38A4 enhances Kupffer cell phagocytosis and suppresses liver metastasis via downregulation of CD24. **a** CD24 protein level in Hepa1-6 cells with SLC38A4 and CD24 concurrent depletion was measured by flow cytometry. **b** In vitro phagocytosis of CFSE-labeled Hepa1-6 cells with SLC38A4 and CD24 concurrent depletion by Kupffer cells was measured by flow cytometry. **c** Representative liver images, hematoxylin–eosin-stained images of liver tissues isolated from C57BL/6 mice at the 14th day after intrasplenic injection with 1 × 10^7^ indicated Hepa1-6 cells, and tumor-replaced areas are shown. Scale bar, 200 µm. **d** CD24 protein level in B16F10 cells with SLC38A4 and CD24 concurrent overexpression was measured by flow cytometry. **e** In vitro phagocytosis of CFSE-labeled B16F10 cells with SLC38A4 and CD24 concurrent overexpression by Kupffer cells was measured by flow cytometry. **f** Representative liver images, hematoxylin–eosin-stained images of liver tissues isolated from C57BL/6 mice at the 13th day after intrasplenic injection with 1 × 10^6^ indicated B16F10 cells, and tumor-replaced areas are shown. Scale bar, 200 µm. Results are shown as mean ± s.d. of *n* = 3 independent experiments (parts **a**, **b**, **d** and **e**), *n* = 6 (part **c**), or *n* = 8 (part **f**) mice in each group by one-way analysis of variance followed by Dunnett’s multiple comparisons test (parts **a** and **b**), Kruskal–Wallis test followed by Dunn’s multiple comparisons test (part **c**), Student’s *t* test (parts **d** and **e**), or Mann–Whitney test (part **f**). ns, not significant. APC allophycocyanin, CFSE carboxyfluorescein succinimidyl ester, MFI mean fluorescence intensity, NC negative control, PE phycoerythrin, SLC solute carrier.

To investigate whether CD24 functionally mediates SLC38A4’s effects on Kupffer cell phagocytosis and liver metastasis, we silenced CD24 expression in SLC38A4-deficient and control Hepa1-6 cells (Fig. 4a). In vitro phagocytosis assays showed that CD24 silencing abrogated the suppressive phagocytosis of Hepa1-6 cells by Kupffer cells caused by SLC38A4 knockdown (Fig. 4b). In vivo liver metastasis assays revealed that CD24 silencing largely abrogated the increased liver metastasis caused by SLC38A4 knockdown (Fig. 4c). Furthermore, we ectopically overexpressed CD24 in SLC38A4-overexpressing and control B16F10 cells (Fig. 4d). In vitro phagocytosis assays showed that CD24 overexpression abrogated the enhanced phagocytosis of B16F10 cells by Kupffer cells caused by SLC38A4 overexpression (Fig. 4e). In vivo liver metastasis assays revealed that CD24 overexpression largely abrogated the suppressive liver metastasis caused by SLC38A4 overexpression (Fig. 4f). To further validate the role of CD24 as the key mediator, we treated SLC38A4-overexpressing and control SW620 cells with CD24 neutralizing antibodies, which enhanced phagocytosis of SW620 cells by Kupffer cells (Supplementary Fig. 6a), and eliminated the differential phagocytosis between SLC38A4-overexpressing and control cells (Supplementary Fig. 6a). Similarly, CD24 blockade abolished the reduced phagocytosis observed in SLC38A4-knockdown cells (Supplementary Fig. 6b). These genetic and pharmacological interventions establish CD24 as the downstream mediator through which SLC38A4 promotes Kupffer cell phagocytosis and suppresses liver metastasis.

### SLC38A4 transcriptionally suppresses CD24 expression through MYC downregulation

**Fig. 5:**
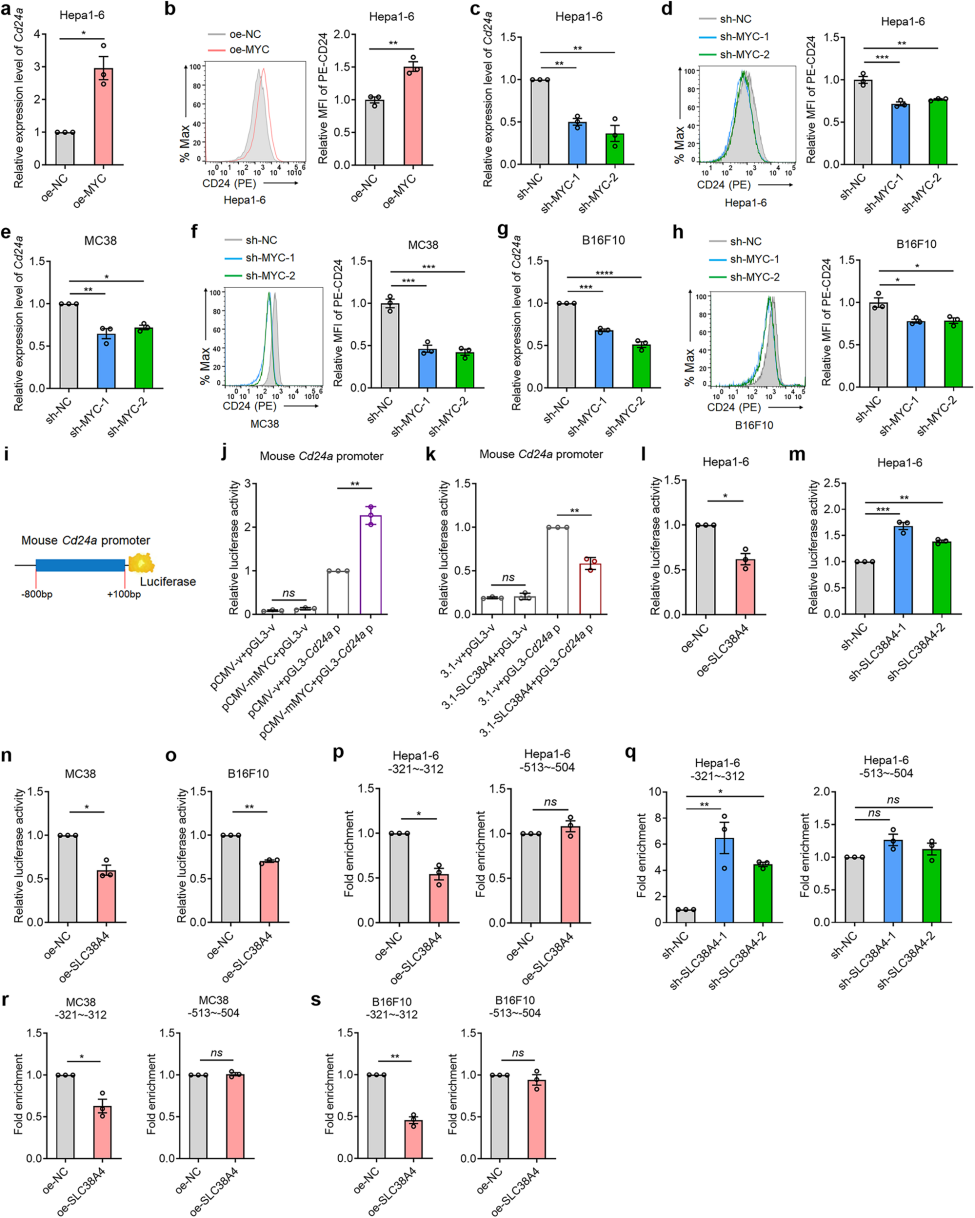
SLC38A4 and MYC suppress *Cd24a* transcription. **a** *Cd24a* mRNA level in Hepa1-6 cells with MYC overexpression or control was measured by quantitative PCR (qPCR). **b** CD24 protein level in Hepa1-6 cells with MYC overexpression or control was measured by flow cytometry. **c** *Cd24a* mRNA level in Hepa1-6 cells with MYC knockdown or control was measured by qPCR. **d** CD24 protein level in Hepa1-6 cells with MYC knockdown or control was measured by flow cytometry. **e** *Cd24a* mRNA level in MC38 cells with MYC knockdown or control was measured by qPCR. **f** CD24 protein level in MC38 cells with MYC knockdown or control was measured by flow cytometry. **g** *Cd24a* mRNA level in B16F10 cells with MYC knockdown or control was measured by qPCR. **h** CD24 protein level in B16F10 cells with MYC knockdown or control was measured by flow cytometry. **i** Schematic of the luciferase reporter containing mouse *Cd24a* promoter. **j** Luciferase activity in 293T cells co-transfected with MYC overexpression vector, luciferase reporter containing *Cd24a* promoter, and pRL-TK vector which encodes Renilla luciferase. Data are shown as the relative ratio of firefly luciferase activity to Renilla luciferase activity. **k** Luciferase activity in 293T cells co-transfected with SLC38A4 overexpression vector, luciferase reporter containing *Cd24a* promoter, and pRL-TK vector. Data are shown as the relative ratio of firefly luciferase activity to Renilla luciferase activity. Luciferase activity in Hepa1-6 cells with SLC38A4 overexpression (part **l**) or knockdown (part **m**), co-transfected with luciferase reporter containing *Cd24a* promoter and pRL-TK vector. Data are shown as the relative ratio of firefly luciferase activity to Renilla luciferase activity. Luciferase activity in MC38 (part **n**) or B16F10 (part **o**) cells with SLC38A4 overexpression or control, co-transfected with luciferase reporter containing *Cd24a* promoter and pRL-TK vector. Data are shown as the relative ratio of firefly luciferase activity to Renilla luciferase activity. CUT&RUN assays followed by qPCR were performed in Hepa1-6 cells with SLC38A4 overexpression (part **p**) or knockdown (part **q**), to detect the binding of MYC to *Cd24a* promoter region covering the −312 site or −504 site. CUT&RUN assays followed by qPCR were performed in MC38 (part **r**) or B16F10 (part **s**) cells with SLC38A4 overexpression or control, to detect the binding of MYC to *Cd24a* promoter region covering the −312 site or −504 site. Results are shown as mean ± s.d. of *n* = 3 independent experiments. *P < 0.05, **P < 0.01, ***P < 0.001, ****P < 0.0001, by Student’s *t* test (parts **a**, **b**, **j**–**l**, **n**–**p**, **r**, and **s**) or one-way analysis of variance followed by Dunnett’s multiple comparisons test (parts **c**–**h**, **m**, and **q**). ns, not significant. c-Myc myelocytomatosis viral oncogene homolog, MYC, MC mouse colon, MFI mean fluorescence intensity, NC negative control, pCMV plasmid cytomegalovirus, PE phycoerythrin, pGL plasmid Green Luciferase, SLC solute carrier.

Next, we investigated the mechanism through which SLC38A4 reduces CD24 expression. The above results showed that SLC38A4 downregulated both mRNA and protein levels of CD24, which suggested that SLC38A4 suppressed CD24 expression at the transcriptional level. Our previous study demonstrated that SLC38A4 reduced the expression of transcription factor MYC^31^. Intriguingly, the online in silico tool JASPAR predicted two conserved MYC-binding sites in both mouse *Cd24a* and human *CD24* promoters (Supplementary Fig. 7a,b). MYC overexpression significantly increased both RNA and protein levels of *Cd24a*/CD24 in Hepa1-6 cells (Fig. 5a,b). Conversely, MYC knockdown decreased RNA and protein levels of *Cd24a*/CD24 in Hepa1-6 cells (Fig. 5c,d). MYC knockdown also decreased RNA and protein levels of *Cd24a*/CD24 in MC38 and B16F10 cells (Fig. 5e–h). Similarly, MYC knockdown in human SNU-398 cells also significantly decreased RNA and protein levels of *CD24*/CD24 (Supplementary Fig. 7c,d). MYC overexpression in human SW620 cells significantly increased RNA and protein levels of *CD24*/CD24 (Supplementary Fig. 7e,f). Likewise, MYC knockdown reduced RNA and protein levels of *CD24*/CD24 in SW620 and A375 cells (Supplementary Fig. 7g–j). These data demonstrate that MYC upregulates CD24 expression.

To mechanistically link SLC38A4-MYC signaling to *CD24* transcriptional regulation, we performed promoter analysis using luciferase reporter assays. We cloned the mouse *Cd24a* or human *CD24* promoter into a luciferase reporter (Fig. 5i and Supplementary Fig. 7k). Dual-luciferase reporter assays showed that MYC overexpression increased, whereas SLC38A4 overexpression suppressed mouse *Cd24a* promoter activity (Fig. 5j,k). Similarly, MYC overexpression increased, whereas SLC38A4 overexpression decreased human *CD24* promoter activity (Supplementary Fig. 7l,m). SLC38A4 overexpression in Hepa1-6 cells decreased *Cd24a* promoter activity (Fig. 5l), whereas SLC38A4 knockdown in Hepa1-6 cells increased *Cd24a* promoter activity (Fig. 5m). Similarly, SLC38A4 overexpression in MC38 and B16F10 cells also decreased *Cd24a* promoter activity (Fig. 5n,o). SLC38A4 overexpression in human tumor cells decreased human *CD24* promoter activity (Supplementary Fig. 7n,o), whereas SLC38A4 knockdown increased human *CD24* promoter activity (Supplementary Fig. 7p,q). CUT&RUN assays with MYC-specific antibody showed that SLC38A4 overexpression in Hepa1-6 cells decreased the binding of MYC to the −312 site, but not to the −504 site of *Cd24a* promoter (Fig. 5p). SLC38A4 knockdown in Hepa1-6 cells increased the binding of MYC to the −312 site but not to the −504 site of *Cd24a* promoter (Fig. 5q). Similarly, SLC38A4 overexpression in B16F10 and MC38 cells also decreased the binding of MYC to the −312 site, but not to the −504 site of *Cd24a* promoter (Fig. 5r,s). In human cells, CUT&RUN assays showed that SLC38A4 overexpression decreased the binding of MYC to the −1173 site, but not to the −864 site of *CD24* promoter (Supplementary Fig. 7r,s). SLC38A4 knockdown increased the binding of MYC to the −1173 site but not to the −864 site of *CD24* promoter (Supplementary Fig. 7t,u). We further mutated the −312 site in mouse *Cd24a* promoter reporter (Supplementary Fig. 8a). Dual-luciferase reporter assays showed that neither overexpression nor knockdown of SLC38A4 regulated the promoter activity of the mutated *Cd24a* promoter (Supplementary Fig. 8b–e). These data suggest that the −312 site of *Cd24a* promoter is critical for the roles of SLC38A4/MYC in *Cd24a* transcription. Moreover, we mutated the −1173 site in human *CD24* promoter reporter (Supplementary Fig. 8f). Dual-luciferase reporter assays showed that mutation of this −1173 site eliminated the role of SLC38A4 in modulating the promoter activity of *CD24* (Supplementary Fig. 8g–j). These data suggest that the –1173 site of *CD24* promoter is critical for the role of SLC38A4/MYC in *CD24* transcription.

**Fig. 6:**
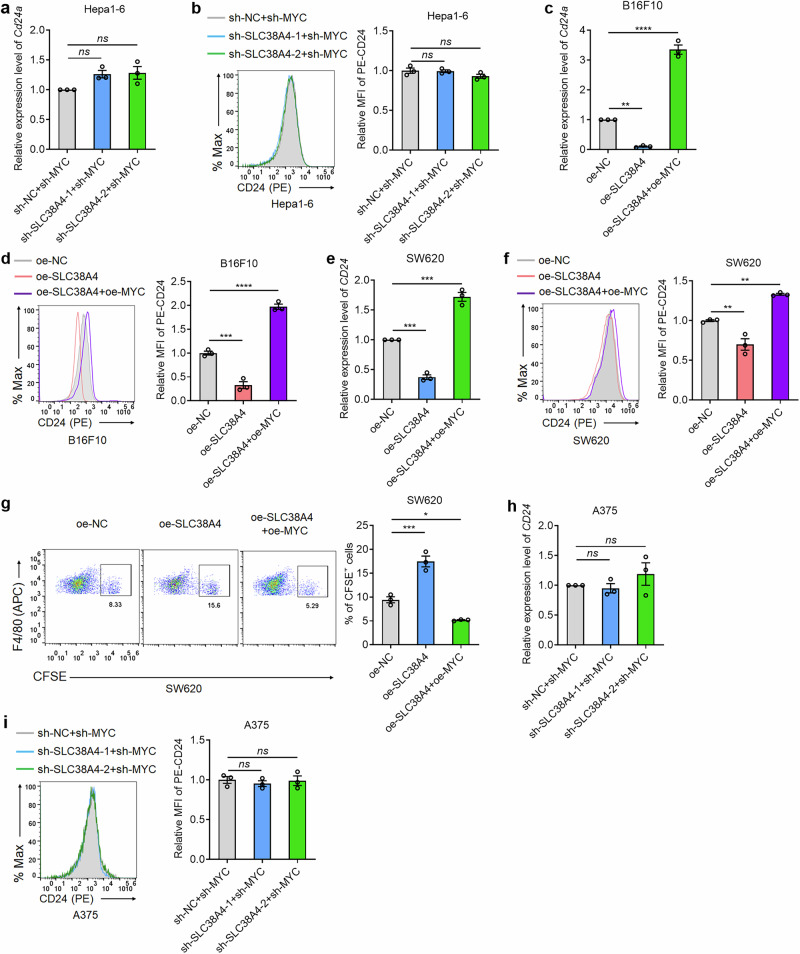
SLC38A4 suppresses CD24 expression through MYC. **a** *Cd24a* mRNA level in Hepa1-6 cells with SLC38A4 and MYC concurrent depletion was measured by quantitative PCR (qPCR). **b** CD24 protein level in Hepa1-6 cells with SLC38A4 and MYC concurrent depletion was measured by flow cytometry. **c** *Cd24a* mRNA level in B16F10 cells with SLC38A4 and MYC concurrent overexpression was measured by qPCR. **d** CD24 protein level in B16F10 cells with SLC38A4 and MYC concurrent overexpression was measured by flow cytometry. **e** *CD24* mRNA level in SW620 cells with SLC38A4 and MYC concurrent overexpression was measured by qPCR. **f** CD24 protein level in SW620 cells with SLC38A4 and MYC concurrent overexpression was measured by flow cytometry. **g** In vitro phagocytosis of CFSE-labeled SW620 cells with SLC38A4 and MYC concurrent overexpression by Kupffer cells was measured by flow cytometry. **h** *CD24* mRNA level in A375 cells with SLC38A4 and MYC concurrent depletion was measured by qPCR. **i** CD24 protein level in A375 cells with SLC38A4 and MYC concurrent depletion was measured by flow cytometry. Results are shown as mean ± s.d. of *n* = 3 independent experiments. *P < 0.05, **P < 0.01, ***P < 0.001, ****P < 0.0001, by one-way analysis of variance followed by Dunnett’s multiple comparisons test. ns, not significant. APC allophycocyanin, CFSE carboxyfluorescein succinimidyl ester, MFI mean fluorescence intensity, NC negative control, PE phycoerythrin, SLC solute carrier.

Next, we evaluated whether MYC was a critical mediator of the role of SLC38A4 in regulating CD24 and phagocytosis. qPCR and flow cytometry assays showed that MYC depletion in SLC38A4-knockdown Hepa1-6 cells abolished the effects of SLC38A4 knockdown on RNA and protein levels of mouse *Cd24a*/CD24 (Fig. 6a,b). MYC overexpression reversed the suppressive effects of SLC38A4 on both RNA and protein levels of mouse *Cd24a*/CD24 (Fig. 6c,d). In human tumor cells, MYC overexpression also reversed the suppressive effects of SLC38A4 on RNA and protein levels of human *CD24*/CD24 (Fig. 6e,f). Furthermore, MYC overexpression reversed the phagocytosis-promoting effects of SLC38A4 (Fig. 6g). Similarly, MYC depletion in SLC38A4-knockdown human tumor cells also abolished the effects of SLC38A4 knockdown on RNA and protein levels of human *CD24*/CD24 (Fig. 6h,i). These results demonstrate that MYC serves as the indispensable molecular switch through which SLC38A4 regulates CD24 expression.

To further validate the regulatory axis of SLC38A4/MYC/CD24, we detected the expression of SLC38A4, MYC, and CD24 using IHC in liver metastatic tissues formed by various mouse and human tumor cells in Fig. 1 and Supplementary Fig. 1. Liver metastatic tissues formed by SLC38A4-overexpresssing Hepa1-6 cells exhibited increased SLC38A4 expression and reduced MYC and CD24 expression, a pattern consistently observed in both C57BL/6 mice and nude mice (Supplementary Fig. 9a–f). Conversely, liver metastatic tissues formed by SLC38A4-knockdown Hepa1-6 cells displayed decreased SLC38A4 expression and increased MYC and CD24 expression (Supplementary Fig. 9g–i). Liver metastatic tissues formed by SLC38A4-overexpresssing MC38 cells also exhibited increased SLC38A4 expression and reduced MYC and CD24 expression in both C57BL/6 (Supplementary Fig. 9j–l) and nude mice (Supplementary Fig. 10a–c). Liver metastatic tissues formed by SLC38A4-overexpresssing B16F10 cells exhibited increased SLC38A4 expression and reduced MYC and CD24 expression, a pattern consistently observed in both C57BL/6 mice and nude mice (Supplementary Fig. 10d–i). Liver metastatic tissues formed by SLC38A4-knockdown SW620 cells also displayed decreased SLC38A4 expression and increased MYC and CD24 expression (Supplementary Fig. 10j–l). Collectively, these data demonstrate that SLC38A4 represses *CD24*/*Cd24a* transcription via MYC.

### SLC38A4 negatively correlates with liver metastasis in humans

**Fig. 7:**
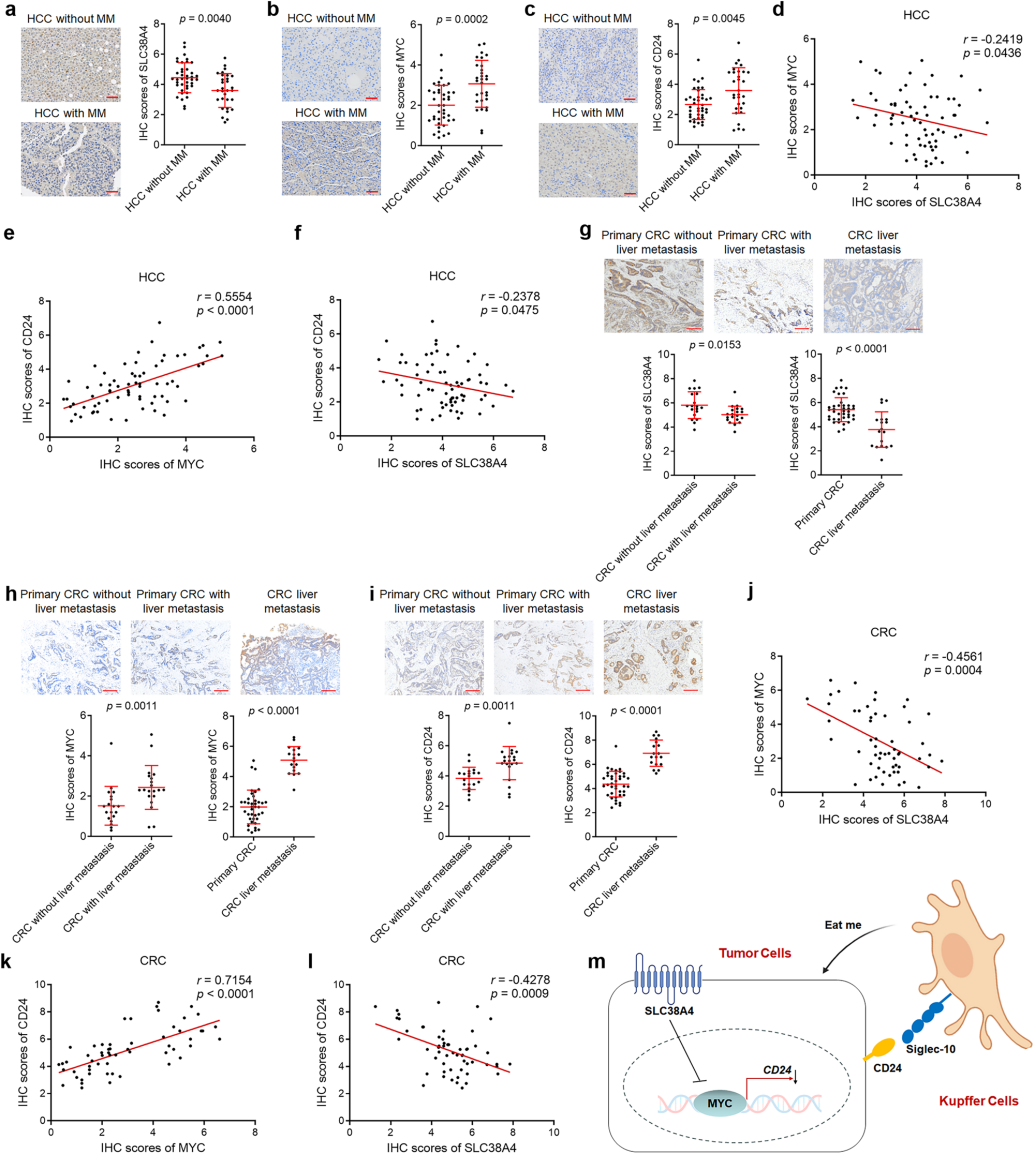
The correlation between SLC38A4, MYC, CD24, and liver metastasis in human tissues. Immunohistochemistry (IHC) staining of SLC38A4 (part **a**), MYC (part **b**), and CD24 (part **c**) in 70 human hepatocellular carcinoma (HCC) tissues, of which 30 had liver micrometastasis (MM) and 40 did not. Scale bar, 50 µm. **d** The correlation between IHC scores of SLC38A4 and MYC in these 70 HCC tissues. *r* = −0.2419, *P* = 0.0436 by Spearman correlation analysis. **e** The correlation between IHC scores of MYC and CD24 in these 70 HCC tissues. *r* = 0.5554, *P* < 0.0001 by Spearman correlation analysis. **f** The correlation between IHC scores of SLC38A4 and CD24 in these 70 HCC tissues. *r* = −0.2378, *P* = 0.0475 by Spearman correlation analysis. IHC staining of SLC38A4 (part **g**), MYC (part **h**), and CD24 (part **i**) in 39 primary colorectal cancer (CRC) tissues, of which 20 had liver metastasis and 19 did not, and 18 had CRC liver metastatic tissues. Scale bar, 200 µm. **j** The correlation between IHC scores of SLC38A4 and MYC in these 57 primary CRC and liver metastatic tissues. *r* = −0.4561, *P* = 0.0004 by Spearman correlation analysis. **k** The correlation between IHC scores of MYC and CD24 in these 57 primary CRC and liver metastatic tissues. *r* = 0.7154, *P* < 0.0001 by Spearman correlation analysis. **l** The correlation between IHC scores of SLC38A4 and CD24 in these 57 primary CRC and liver metastatic tissues. *r* = −0.4278, *P* = 0.0009 by Spearman correlation analysis. **m** Schematic of the roles of SLC38A4 in enhancing Kupffer cell phagocytosis through MYC/CD24 axis. For parts **a**–**c** and **g**–**i** results are shown as median with interquartile range. *P*-values were calculated using the Mann–Whitney test. SLC solute carrier.

To analyze clinical relevance of SLC38A4/MYC/CD24 regulatory axis, we measured SLC38A4, MYC, and CD24 expression in 70 HCC tissues using IHC. Among these samples, 30 had liver micrometastases (MM) and 40 did not. IHC results showed that SLC38A4 expression was significantly lower in HCC tissues with liver MM than in those without liver MM (Fig. 7a). Conversely, MYC and CD24 expression were higher in HCC tissues with liver MM than in those without liver MM (Fig. 7b,c). Furthermore, SLC38A4 expression was negatively correlated with MYC and CD24 expression, whereas MYC expression was positively correlated with CD24 in these 70 HCC tissues (Fig. 7d–f). Analysis of the Cancer Genome Atlas Program data confirmed that *SLC38A4* RNA levels were negatively correlated with *MYC* and *CD24* RNA levels, whereas *MYC* and *CD24* RNA levels were positively correlated in HCC tissues (Supplementary Fig. 11a–c). Similarly, GEO database^35^ analysis showed that *SLC38A4* RNA levels were negatively correlated with *MYC* and *CD24* RNA levels, whereas *MYC* and *CD24* RNA levels were positively correlated in metastatic HCC tissues (Supplementary Fig. 11d–f).

Next, IHC was performed to measure SLC38A4 expression in 39 CRC tissues, of which 20 had liver metastasis and 19 did not. The results showed that SLC38A4 expression was lower in CRC tissues with liver metastasis compared with those without liver metastasis (Fig. 7g). Additionally, we collected 18 CRC liver metastatic tissues and measured SLC38A4 expression in these liver metastatic tissues using IHC. The results showed that SLC38A4 expression was reduced in liver metastatic tissues compared with primary CRC tissues (Fig. 7g). Conversely, MYC and CD24 expressions were higher in CRC tissues with liver metastasis than in CRC tissues without liver metastasis and were also elevated in liver metastatic tissues relative to primary CRC tissues (Fig. 7h,i). Furthermore, SLC38A4 expression showed a negative correlation with MYC and CD24, whereas MYC and CD24 expressions were positively correlated in these 57 primary and metastatic CRC tissues (Fig. 7j–l). Analysis of the GEO database^36^ showed that *SLC38A4* RNA levels were negatively correlated with *MYC* and *CD24* RNA levels, whereas *MYC* and *CD24* RNA levels were positively correlated in CRC liver metastatic tissues (Supplementary Fig. 11g–i). Taken together, these data suggest that SLC38A4 negatively correlates with MYC, CD24, and liver metastasis in humans.

## Discussion

The role of macrophages in metastasis is obscured by the diversity between and within various tissue microenvironments^37–41^. Kupffer cells, which are constitutively resident in the liver, have crucial roles in innate immune defense against a wide array of microorganisms and tumor cells^42^. In this study, we identified SLC38A4 as an inducer of Kupffer cell phagocytosis of various tumor cells, including CRC, HCC, and melanoma cells, leading to the suppression of tumor liver metastasis (Fig. 7m).

Our previous study showed that SLC38A4 reduces MYC expression by inhibiting Wnt/β-catenin signaling^31^. In this study, we identified MYC as a transcriptional activator of CD24. MYC binds to the promoter region of *CD24*/*Cd24a* and activates the transcription of *CD24*/*Cd24a*. By reducing MYC, SLC38A4 suppresses the transcription of *CD24*/*Cd24a*, leading to the downregulation of *CD24*/*Cd24a* mRNA and CD24 protein levels in tumor cells. Previous reports identified CD24 expressed on tumor cells as a don’t eat me signal for macrophages^27,43^. Here, we further showed that CD24 expressed on tumor cells also functions as a don’t eat me signal for Kupffer cells. By suppressing CD24 expression, SLC38A4 promotes the in vivo and in vitro phagocytosis of various tumor cells by Kupffer cells. SLC38A4 did not modulate other phagocytosis signals expressed on tumor cells, such as CD47 and PD-L1. Depletion of *CD24*/*Cd24a* or CD24 blockade abolished the role of SLC38A4 in Kupffer cell phagocytosis, suggesting that CD24 is a critical mediator of the roles of SLC38A4 in Kupffer cell phagocytosis.

In vivo liver metastasis assays showed that overexpression of SLC38A4 significantly suppressed, whereas knockdown of SLC38A4 promoted liver metastasis of various tumor cells. Our previous study found that SLC38A4 inhibits HCC cell proliferation^31^. To exclude the potential effects of cell proliferation on liver metastasis, we detected liver metastases as early as 12 h after intrasplenic injection using bioluminescence imaging, at which time cell proliferation had no significant effect on cell number. Bioluminescence imaging results showed that SLC38A4 significantly suppressed liver metastasis as early as 12 h after intrasplenic injection. These data suggest that in addition to cell proliferation, other factors also mediate the role of SLC38A4 in liver metastasis. Considering that SLC38A4 suppresses liver metastasis in both immunocompetent C57BL/6 mice and nude mice that lack T cells, the role of SLC38A4 in liver metastasis is not dependent on T cells. Kupffer cells reside alongside the liver sinusoid and phagocytize invading tumor cells early after tumor cells reach the liver^44^. Therefore, we evaluated whether the suppressive roles of SLC38A4 in liver metastasis were dependent on Kupffer cells. Our results showed that depletion of Kupffer cells significantly promoted liver metastasis. Moreover, depletion of Kupffer cells largely abolished the role of SLC38A4 in liver metastasis. These data suggest that Kupffer cell phagocytosis induced by SLC38A4 mediates the suppressive role of SLC38A4 in tumor liver metastasis.

Targeted immunomodulation aimed at reactivating innate immune cells, with a focus on macrophages, shows significant potential for complementing adaptive immunotherapy^45,46^. However, in the context of solid tumors, there is a dearth of effective and safe therapeutic agents for promoting macrophage phagocytosis^47^. This study suggests that the SLC38A4/MYC/CD24 axis may be a potential therapeutic target for liver metastasis through enhancing Kupffer cell phagocytosis.

In conclusion, our study demonstrated that SLC38A4 suppresses tumor liver metastasis via enhancing phagocytosis of tumor cells by Kupffer cells. CD24 is verified as a pro-phagocytic signal for Kupffer cells, not only for macrophages. MYC directly activates *CD24* transcription through binding to the *CD24* promoter. SLC38A4 suppresses the expression of *CD24* through downregulating MYC. SLC38A4 promotes Kupffer cell phagocytosis and suppresses liver metastasis through decreasing CD24 expression in tumor cells. The SLC38A4/MYC/CD24 regulatory axis represents a potential therapeutic target for liver metastasis.

## Supplementary information

Supplementary Information

raw western blots

## Data Availability

The data presented in this study are available upon reasonable request from the corresponding authors.

## References

[1] Chaffer, C. L. & Weinberg, R. A. A perspective on cancer cell metastasis. *Science* **331**, 1559–1564 (2011). 21436443 10.1126/science.1203543

[2] Tsilimigras, D. I. et al. Liver metastases. *Nat Rev Dis Primers* **7**, 27 (2021). 33859205 10.1038/s41572-021-00261-6

[3] Kim, M. S., Lee, W. S. & Jin, W. TrkB inhibition of DJ-1 degradation promotes the growth and maintenance of cancer stem cell characteristics in hepatocellular carcinoma. *Cell Mol Life Sci* **80**, 303 (2023). 37749450 10.1007/s00018-023-04960-z PMC10520132

[4] LeFort, K. R., Rungratanawanich, W. & Song, B. J. Contributing roles of mitochondrial dysfunction and hepatocyte apoptosis in liver diseases through oxidative stress, post-translational modifications, inflammation, and intestinal barrier dysfunction. *Cell Mol Life Sci* **81**, 34 (2024). 38214802 10.1007/s00018-023-05061-7 PMC10786752

[5] Mielgo, A. & Schmid, M. C. Liver tropism in cancer: the hepatic metastatic niche.*Cold Spring Harb Perspect Med* **10**, a037259 (2020). 31548227 10.1101/cshperspect.a037259 PMC7050581

[6] Hashemi, M. et al. Deciphering STAT3 signaling potential in hepatocellular carcinoma: tumorigenesis, treatment resistance, and pharmacological significance. *Cell Mol Biol Lett* **28**, 33 (2023). 37085753 10.1186/s11658-023-00438-9 PMC10122325

[7] Skrip, L. M. et al. Viscoelastic properties of colorectal liver metastases reflect tumour cell viability. *J Transl Med* **22**, 774 (2024). 39152426 10.1186/s12967-024-05559-z PMC11328469

[8] Mariani, P. et al. Immunohistochemical characterisation of the immune landscape in primary uveal melanoma and liver metastases. *Br J Cancer* **129**, 772–781 (2023). 37443346 10.1038/s41416-023-02331-w PMC10449826

[9] Gui, M. et al. Integrative single-cell transcriptomic analyses reveal the cellular ontological and functional heterogeneities of primary and metastatic liver tumors. *J Transl Med* **22**, 206 (2024). 38414027 10.1186/s12967-024-04947-9 PMC10898050

[10] Guilliams, M. & Scott, C. L. Liver macrophages in health and disease. *Immunity* **55**, 1515–1529 (2022). 36103850 10.1016/j.immuni.2022.08.002

[11] Bennett, H. et al. Discrimination of cell-intrinsic and environment-dependent effects of natural genetic variation on Kupffer cell epigenomes and transcriptomes. *Nat Immunol* **24**, 1825–1838 (2023). 37735593 10.1038/s41590-023-01631-w PMC10602851

[12] Li, P., He, K., Li, J., Liu, Z. & Gong, J. The role of Kupffer cells in hepatic diseases. *Mol Immunol* **85**, 222–229 (2017). 28314211 10.1016/j.molimm.2017.02.018

[13] Peng, B. et al. Intrahepatic macrophage reprogramming associated with lipid metabolism in hepatitis B virus-related acute-on-chronic liver failure. *J Transl Med* **21**, 419 (2023). 37380987 10.1186/s12967-023-04294-1 PMC10303321

[14] Liu, K. et al. Dynamic YAP expression in the non-parenchymal liver cell compartment controls heterologous cell communication. *Cell Mol Life Sci* **81**, 115 (2024). 38436764 10.1007/s00018-024-05126-1 PMC10912141

[15] Lin, H. et al. Gram-negative bacteria-driven increase of cytosolic phospholipase A2 leads to activation of Kupffer cells. *Cell Mol Life Sci* **82**, 22 (2024). 39725773 10.1007/s00018-024-05451-5 PMC11671446

[16] Park, J. S. et al. A1AT dysregulation of metabolically stressed hepatocytes by Kupffer cells drives MASH and fibrosis. *Exp Mol Med* **57**, 450–465 (2025). 39939782 10.1038/s12276-025-01408-1 PMC11873038

[17] Lee, E. H. et al. Loss of SREBP-1c ameliorates iron-induced liver fibrosis by decreasing lipocalin-2. *Exp Mol Med* **56**, 1001–1012 (2024). 38622198 10.1038/s12276-024-01213-2 PMC11058876

[18] Deng, Z. et al. The nuclear factor ID3 endows macrophages with a potent anti-tumour activity. *Nature* **626**, 864–873 (2024). 38326607 10.1038/s41586-023-06950-4 PMC10881399

[19] Li, J. et al. The ligation between ERMAP, galectin-9 and dectin-2 promotes Kupffer cell phagocytosis and antitumor immunity. *Nat Immunol* **24**, 1813–1824 (2023). 37813965 10.1038/s41590-023-01634-7

[20] Kimura, Y. et al. The innate immune receptor Dectin-2 mediates the phagocytosis of cancer cells by Kupffer cells for the suppression of liver metastasis. *Proc Natl Acad Sci USA* **113**, 14097–14102 (2016). 27872290 10.1073/pnas.1617903113 PMC5150405

[21] Lu, W. P. et al. m(6)A-modified MIR670HG suppresses tumor liver metastasis through enhancing Kupffer cell phagocytosis. *Cell Mol Life Sci* **82**, 185 (2025). 40293529 10.1007/s00018-025-05700-1 PMC12037464

[22] Feng, M. et al. Phagocytosis checkpoints as new targets for cancer immunotherapy. *Nat Rev Cancer* **19**, 568–586 (2019). 31462760 10.1038/s41568-019-0183-z PMC7002027

[23] Chen, J. et al. SLAMF7 is critical for phagocytosis of haematopoietic tumour cells via Mac-1 integrin. *Nature* **544**, 493–497 (2017). 28424516 10.1038/nature22076 PMC5565268

[24] Feng, M. et al. Programmed cell removal by calreticulin in tissue homeostasis and cancer. *Nat Commun* **9**, 3194 (2018). 30097573 10.1038/s41467-018-05211-7 PMC6086865

[25] Oldenborg, P. A., Gresham, H. D. & Lindberg, F. P. CD47-signal regulatory protein alpha (SIRPalpha) regulates Fcgamma and complement receptor-mediated phagocytosis. *J Exp Med* **193**, 855–862 (2001). 11283158 10.1084/jem.193.7.855 PMC2193364

[26] Gordon, S. R. et al. PD-1 expression by tumour-associated macrophages inhibits phagocytosis and tumour immunity. *Nature* **545**, 495–499 (2017). 28514441 10.1038/nature22396 PMC5931375

[27] Barkal, A. A. et al. CD24 signalling through macrophage Siglec-10 is a target for cancer immunotherapy. *Nature* **572**, 392–396 (2019). 31367043 10.1038/s41586-019-1456-0 PMC6697206

[28] Barkal, A. A. et al. Engagement of MHC class I by the inhibitory receptor LILRB1 suppresses macrophages and is a target of cancer immunotherapy. *Nat Immunol* **19**, 76–84 (2018). 29180808 10.1038/s41590-017-0004-z PMC5832354

[29] Chao, M. P. et al. Anti-CD47 antibody synergizes with rituximab to promote phagocytosis and eradicate non-Hodgkin lymphoma. *Cell* **142**, 699–713 (2010). 20813259 10.1016/j.cell.2010.07.044 PMC2943345

[30] Zeng, X., Ward, S. E., Zhou, J. & Cheng, A. S. L. Liver immune microenvironment and metastasis from colorectal cancer-pathogenesis and therapeutic perspectives. *Cancers* (*Basel*) **13**, 2418 (2021). 10.3390/cancers13102418 PMC8156220 34067719

[31] Li, J. et al. SLC38A4 functions as a tumour suppressor in hepatocellular carcinoma through modulating Wnt/beta-catenin/MYC/HMGCS2 axis. *Br J Cancer* **125**, 865–876 (2021). 34274945 10.1038/s41416-021-01490-y PMC8438090

[32] Khosroshahi, E. M. et al. Determining expression changes of ANO7 and SLC38A4 membrane transporters in colorectal cancer. *Heliyon* **10**, e34464 (2024). 39114022 10.1016/j.heliyon.2024.e34464 PMC11305260

[33] Yuan, J. H. et al. A long noncoding RNA activated by TGF-beta promotes the invasion-metastasis cascade in hepatocellular carcinoma. *Cancer Cell* **25**, 666–681 (2014). 24768205 10.1016/j.ccr.2014.03.010

[34] Matsumura, H. et al. Kupffer cells decrease metastasis of colon cancer cells to the liver in the early stage. *Int J Oncol* **45**, 2303–2310 (2014). 25231346 10.3892/ijo.2014.2662

[35] Ye, Q. H. et al. Predicting hepatitis B virus-positive metastatic hepatocellular carcinomas using gene expression profiling and supervised machine learning. *Nat Med* **9**, 416–423 (2003). 12640447 10.1038/nm843

[36] Moosavi, S. H. et al. Molecular prognostic factors for liver transplantation of unresectable metastatic colorectal cancer. *Br J Surg* **112**, znaf072 (2025). 10.1093/bjs/znaf072 PMC12000646 40235343

[37] Mass, E. et al. Specification of tissue-resident macrophages during organogenesis. *Science* **353**, aaf4238 (2016). 10.1126/science.aaf4238 PMC5066309 27492475

[38] Masui, H. et al. Synergistic antitumor activity by dual blockade of CCR1 and CXCR2 expressed on myeloid cells within the tumor microenvironment. *Br J Cancer* **131**, 63–76 (2024). 38750114 10.1038/s41416-024-02710-x PMC11231281

[39] Zhao, L. et al. LINC00330/CCL2 axis-mediated ESCC TAM reprogramming affects tumor progression. *Cell Mol Biol Lett* **29**, 77 (2024). 38769475 10.1186/s11658-024-00592-8 PMC11103861

[40] Geraldo, L. H. et al. CCL21-CCR7 signaling promotes microglia/macrophage recruitment and chemotherapy resistance in glioblastoma. *Cell Mol Life Sci* **80**, 179 (2023). 37314567 10.1007/s00018-023-04788-7 PMC10267017

[41] Yan, Q. et al. Tumor-associated macrophage-derived exosomal miR21-5p promotes tumor angiogenesis by regulating YAP1/HIF-1alpha axis in head and neck squamous cell carcinoma. *Cell Mol Life Sci* **81**, 179 (2024). 38602536 10.1007/s00018-024-05210-6 PMC11009780

[42] Meterissian, S. H., Toth, C. A., Steele, G. Jr & Thomas, P. Kupffer cell/tumor cell interactions and hepatic metastasis in colorectal cancer. *Cancer Lett* **81**, 5–12 (1994). 7517341 10.1016/0304-3835(94)90157-0

[43] Zhang, W. et al. An in-situ peptide-antibody self-assembly to block CD47 and CD24 signaling enhances macrophage-mediated phagocytosis and anti-tumor immune responses. *Nat Commun* **15**, 5670 (2024). 38971872 10.1038/s41467-024-49825-6 PMC11227529

[44] Thomas, S. K. et al. Kupffer cells prevent pancreatic ductal adenocarcinoma metastasis to the liver in mice. *Nat Commun* **14**, 6330 (2023). 37816712 10.1038/s41467-023-41771-z PMC10564762

[45] Kubli, S. P., Berger, T., Araujo, D. V., Siu, L. L. & Mak, T. W. Beyond immune checkpoint blockade: emerging immunological strategies. *Nat Rev Drug Discov* **20**, 899–919 (2021). 33686237 10.1038/s41573-021-00155-y

[46] Dong, X. et al. Efficacy evaluation of chimeric antigen receptor-modified human peritoneal macrophages in the treatment of gastric cancer. *Br J Cancer* **129**, 551–562 (2023). 37386139 10.1038/s41416-023-02319-6 PMC10403530

[47] Zhang, X. et al. Reprogramming tumour-associated macrophages to outcompete cancer cells. *Nature* **619**, 616–623 (2023). 37380769 10.1038/s41586-023-06256-5 PMC10719927

