# Supplementary material for: SLC38A4 promotes Kupffer cell phagocytosis and suppresses tumor liver metastasis
Source: Exp Mol Med. 2026 May 1;58(5):1425–38. doi: 10.1038/s12276-026-01703-5 (PMC13234026; doi:10.1038/s12276-026-01703-5)
Supplement: Supplementary file 1 — Supplementary Information [file 12276_2026_1703_MOESM1_ESM.pdf]

Supplementary Information

Supplementary Table 1 Primer and shRNA sequences used in this study.

**qPCR primer**	**Forward primer (5'-3')**	**Reverse primer (5'-3')**
Slc38a4	GCTCTACTCGGTTCACCTTT	TGATTACTTCAGGCAGTTCGT
SLC38A4	TGCCGCCCTCTTTGGTTA	AAGGACTGCCAGGCGAAC
Cd24a	CTTCGTCTTCCCCTTCTCA	TGGTGGTAGCGTTACTTGG
CD24	CTGCTGGCACTGCTCCTACC	CGAAGAGACTGGCTGTTGACT
Cd47	ACACTGACCAAGGATCAGCC	ACCAAACTTTCCCCAGAACA
CD47	CCCAATTTTTGCTATACTCCTG	CCAACAATGACAATGACAGTGA
Cd274	GTGCGGACTACAAGCGAATC	GTGGCGTTGACCCTCAGACT
CD274	CAGCACACTGAGAATCAACAC	GCTACACCAAGGCATAATAAG
Mouse *β-actin*	CAGCCTTCCTTCTTGGGTAT	TGATCTTGATCTTCATGGTGC
Human *β-actin*	GGGAAATCGTGCGTGACATTAAG	TGTGTTGGCGTACAGGTCTTTG
**Plasmid construction primer**	**Forward primer (5'-3')**	**Reverse primer (5'-3')**
Mouse oe-SLC38A4	CTTGGTACCGAGCTCGGATCCATGGACCCCATGGAACTGAA	CTGGATATCTGCAGAATTCCAGTGGTGATTGGGATTCGGCG
Mouse oe-MYC	ATCCAAGCTTCTGCAGGAATTCATGCCCCTCAACGTGAACTTCACCAA	ACCGGGCCCACTAGTTCTAGATTATGCACCAGAGTTTCGAAGCTG
Human oe-MYC	ATCCAAGCTTCTGCAGGAATTCATGCCCCTCAACGTTAGCTTCACCA	ACCGGGCCCACTAGTTCTAGATTACGCACAAGAGTTCCGTAGCTG
Mouse oe-CD24	GTACCGAGCTCGGATCCATGTCCCTAGGGCTTTTTGCC	CTGGATATCTGCAGAATTCCAACAGTAGAGATGTAGAAGAGAGAG

Mouse sh-SLC38A4-1	TGCAGCCAATTTGCTAATGAATTCAAGAGATTCATTAGCAAATTGGCTGTTTTTTC	TCGAGAAAAAACAGCCAATTTGCTAATGAATCTCTTGAATTCATTAGCAAATTGGCTGCA
Mouse sh-SLC38A4-2	TGCAAGGTCTACACATTTGATTCAAGAGATCAAATGTGTAGACCTTGCTTTTTTC	TCGAGAAAAAAGCAAGGTCTACACATTTGATCTCTTGAATCAAATGTGTAGACCTTGCA
Mouse sh-NC	TGTTCTCCGAACGTGTCACGTTTCAAGAGAACGTGACACGTTCGGAGAACTTTTTTC	TCGAGAAAAAAGTTCTCCGAACGTGTCACGTTCTCTTGAAACGTGACACGTTCGGAGAACA
Mouse sh-CD24	GATCCGATTTACTGCAACCAAACATCTTCAAGAGAGATGTTTGGTTGCAGTAAATCTTTTTTG	AATTCAAAAAAGATTTACTGCAACCAAACATCTCTCTTGAAGATGTTTGGTTGCAGTAAATCG
Mouse sh-MYC-1	CACCGTGGAGATGATGACCGAGTTACTTCAAGAGAGTAACTCGGTCATCATCTCCATTTTTTG	GATCCAAAAAATGGAGATGATGACCGAGTTACTCTCTTGAAGTAACTCGGTCATCATCTCCAC
Mouse sh-MYC-2	CACCGTGAGGATATCTGGAAGAAATTTCAAGAGAATTTCTTCCAGATATCCTCACTTTTTTG	GATCCAAAAAAGTGAGGATATCTGGAAGAAATTCTCTTGAAATTTCTTCCAGATATCCTCAC
Human sh-MYC-1	CACCGCTCGGTGCAGCCGTATTTCTATTCAAGAGATAGAAATACGGCTGCACCGAGTTTTTTG	GATCCAAAAAACTCGGTGCAGCCGTATTTCTATCTCTTGAATAGAAATACGGCTGCACCGAGC
Human sh-MYC-2	CACCGTTCTCTCCGTCCTCGGATTCTTTCAAGAGAAGAATCCGAGGACGGAGAGAATTTTTTG	GATCCAAAAAATTCTCTCCGTCCTCGGATTCTTCTCTTGAAAGAATCCGAGGACGGAGAGAAC
Mouse *Cd24a* promoter	CGTGCTAGCCCGGGCAACTGAGGGGCAGAAAAAAC	CCGGAATGCCAAGCTTGTTCCTCTAGTGCTGCATC
Human *CD24* promoter	CGTGCTAGCCCGGGCTGCAATCAATCTGTTTCAAAATC	CCGGAATGCCAAGCTTAAAAGCCTCAGTTCCTTCATCTA
Mouse *Cd24a* promoter mutation-Fragment1	AACTGAGGGGCAGAAAAAAC	TACAGACCTTAAAGCAAAAC
Mouse *Cd24a* promoter mutation-Fragment2	GCTAAACTTTAAATAGGAAGGTCCT	GTTCCTCTAGTGCTGCATC
Human *CD24* promoter mutation-Fragment1	TGCAATCAATCTGTTTCAAAATC	CCAATTTCCATTTTATAGAGAAAG

Human *CD24* promoter mutation-Fragment2	CAACAAAAAGTTCAGGATAACTGG	AAAAGCCTCAGTTCCTTCATCTA
**qPCR primer for CUT&RUN**	**Forward primer (5’-3’)**	**Reverse primer (5’-3’)**
Mouse *Cd24a* (-321~-312)	CCACCGTTGCTAGGTTTGCTT	GGAATCTTTCTTTCGACCTTCTTTT
Mouse *Cd24a* (-513~-504)	CTGGGCACAGAACGACCTTT	TTACCAAGTCAGCCACAACC
Human *CD24* (-1182~-1173)	CTGTTTCAAAATCATTCTATACC	CTAACAAATTGAGACTCTTACCA
Human *CD24* (-873~-864)	GTAGAGACGGGGTTTCATCA	AGTGGCTCACGCCTATAATC

Supplementary Figure 1

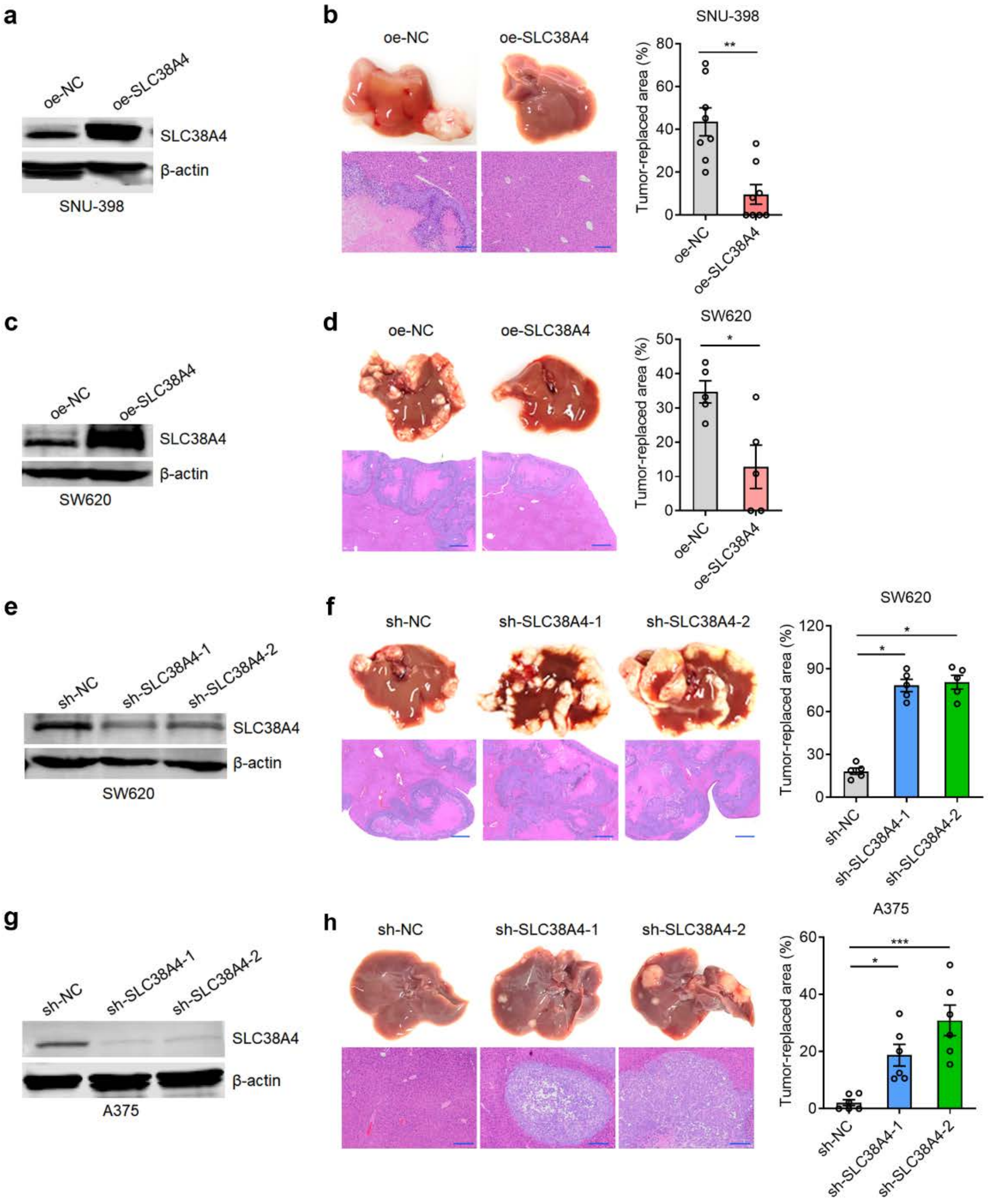

Supplementary Fig. 1 SLC38A4 suppresses liver metastasis of human tumor cells. a SLC38A4 protein levels in SNU-398 cells with SLC38A4 stable overexpression or control was measured by western blot. **b** Representative liver images, HE-stained images of liver tissues isolated from nude mice at the 49th day after intrasplenic injection with 1×10^7 indicated SNU-398 cells, and tumor-replaced areas are shown. Scale bars, 200 μm. **c** SLC38A4 protein levels in SW620 cells with

SLC38A4 stable overexpression or control was measured by western blot. **d** Representative liver images, HE-stained images of liver tissues isolated from nude mice at the 56th day after intrasplenic injection with 1×10^7 indicated SW620 cells, and tumor-replaced areas are shown. Scale bars, 500 µm. **e** SLC38A4 protein levels in SW620 cells with SLC38A4 stable knockdown or control was measured by western blot. **f** Representative liver images, HE-stained images of liver tissues isolated from nude mice at the 56th day after intrasplenic injection with 5×10^6 indicated SW620 cells, and tumor-replaced areas are shown. Scale bars, 500 µm. **g** SLC38A4 protein levels in A375 cells with SLC38A4 stable knockdown or control was measured by western blot. **h** Representative liver images, HE-stained images of liver tissues isolated from nude mice at the 35th day after intrasplenic injection with 1×10^6 indicated A375 cells, and tumor-replaced areas are shown. Scale bars, 200 µm. Results are shown as mean ± s.d. of n = 8 (**b**), n = 5 (**d**, **f**), or n = 6 (**h**) mice in each group. *$p < 0.05$, **$p < 0.01$, ***$p < 0.001$ by Mann-Whitney test (**b**, **d**) or Kruskal-Wallis test followed by Dunn's multiple comparisons test (**f**, **h**).

Supplementary Figure 2

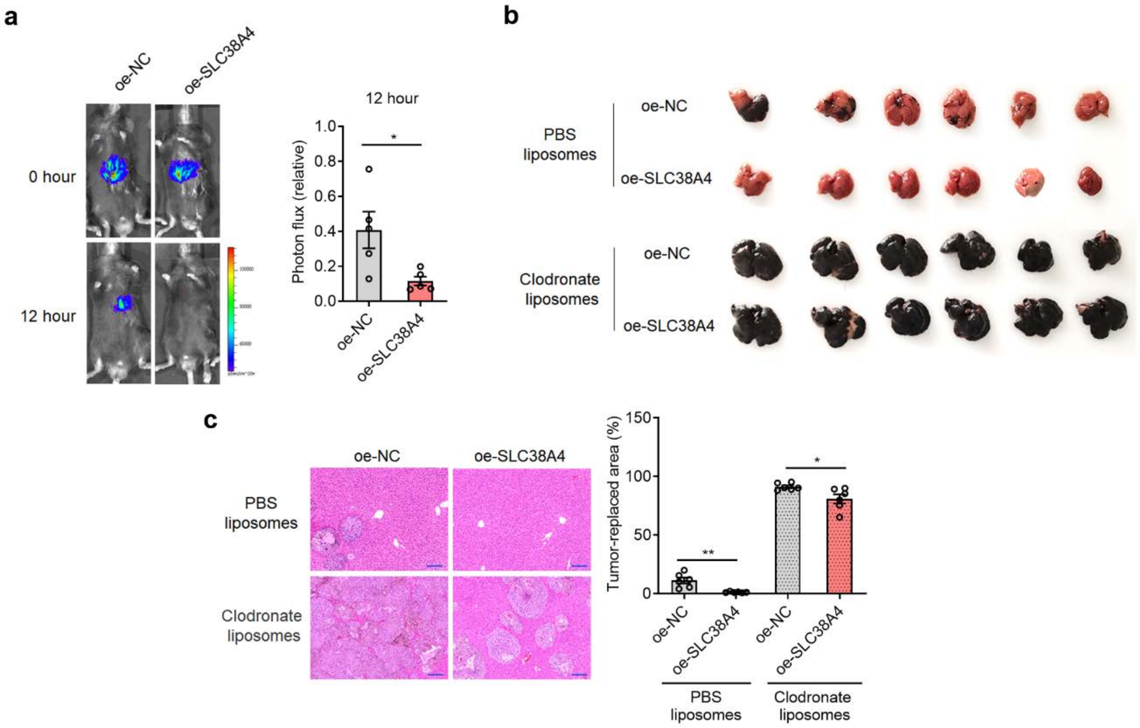

Supplementary Fig. 2 Kupffer cells mediates the suppressive role of SLC38A4 in tumor liver metastasis. a Luciferase signal intensity of C57BL/6 mice at 12 hours after intrasplenic injection with 1×10^6 indicated B16F10 cells. **b** 1×10^6 B16F10 cells with SLC38A4 overexpression or control were intrasplenically injected into C57BL/6 mice treated with control liposomes or clodronate liposomes on day -1. At the 7th day after injection, the mice were sacrificed. Liver images are shown. **c** Representative HE-stained images of liver tissues from the mice from **b**, and tumor-replaced areas are shown. Scale bars, 100 µm. Results are shown as mean ± s.d. of n = 5 (**a**) or n = 6 (**b**, **c**) mice in each group. *$p < 0.05$, **$p < 0.01$ by Mann-Whitney test.

Supplementary Figure 3

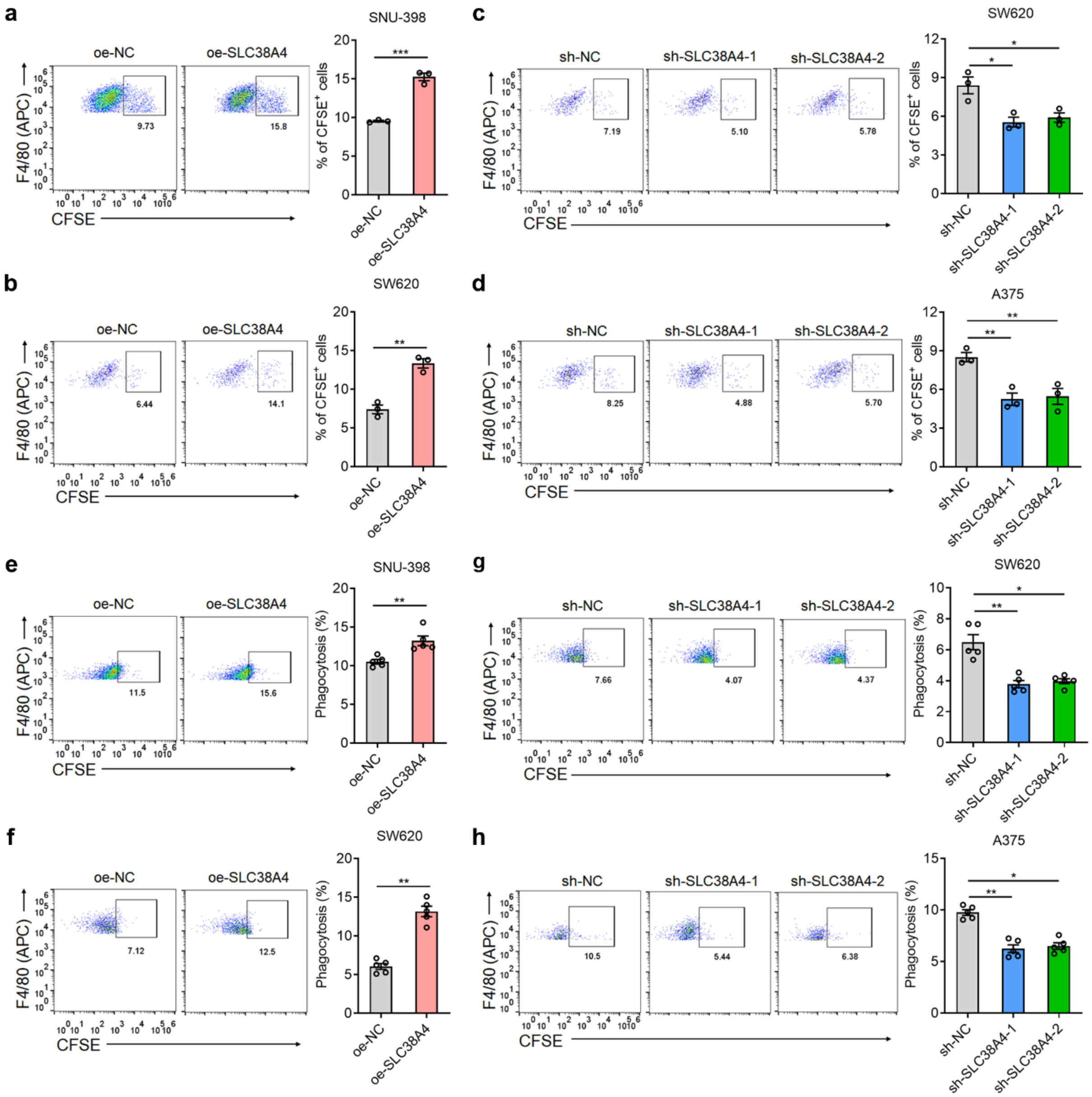

Supplementary Fig. 3 SLC38A4 enhances phagocytosis of human tumor cells by Kupffer cells. a In vitro phagocytosis of CFSE-labeled SNU-398 cells with SLC38A4 overexpression or control by Kupffer cells was measured by flow cytometry. **b** In vitro phagocytosis of CFSE-labeled SW620 cells with SLC38A4 overexpression or control by Kupffer cells was measured by flow cytometry. **c** In vitro phagocytosis of CFSE-labeled SW620 cells with SLC38A4 knockdown or control by

Kupffer cells was measured by flow cytometry. **d** In vitro phagocytosis of CFSE-labeled A375 cells with SLC38A4 knockdown or control by Kupffer cells was measured by flow cytometry. **e** In vivo phagocytosis of CFSE-labeled SNU-398 cells with SLC38A4 overexpression or control by Kupffer cells was assessed by flow cytometry 12h later after intrasplenic inoculation. **f** In vivo phagocytosis of CFSE-labeled SW620 cells with SLC38A4 overexpression or control by Kupffer cells was assessed by flow cytometry 12h later after intrasplenic inoculation. **g** In vivo phagocytosis of CFSE-labeled SW620 cells with SLC38A4 knockdown or control by Kupffer cells was assessed by flow cytometry 12h later after intrasplenic inoculation. **h** In vivo phagocytosis of CFSE-labeled A375 cells with SLC38A4 knockdown or control by Kupffer cells was assessed by flow cytometry 12h later after intrasplenic inoculation. Results are shown as mean ± s.d. of n = 3 independent experiments (**a**-**d**) or n = 5 mice in each group (**e**-**h**). *$p < 0.05$, **$p < 0.01$, ***$p < 0.001$ by Student's *t* test (**a**, **b**), one-way ANOVA followed by Dunnett's multiple comparisons test (**c**, **d**), Mann-Whitney test (**e**, **f**) or Kruskal-Wallis test followed by Dunn's multiple comparisons test (**g**, **h**).

Supplementary Figure 4

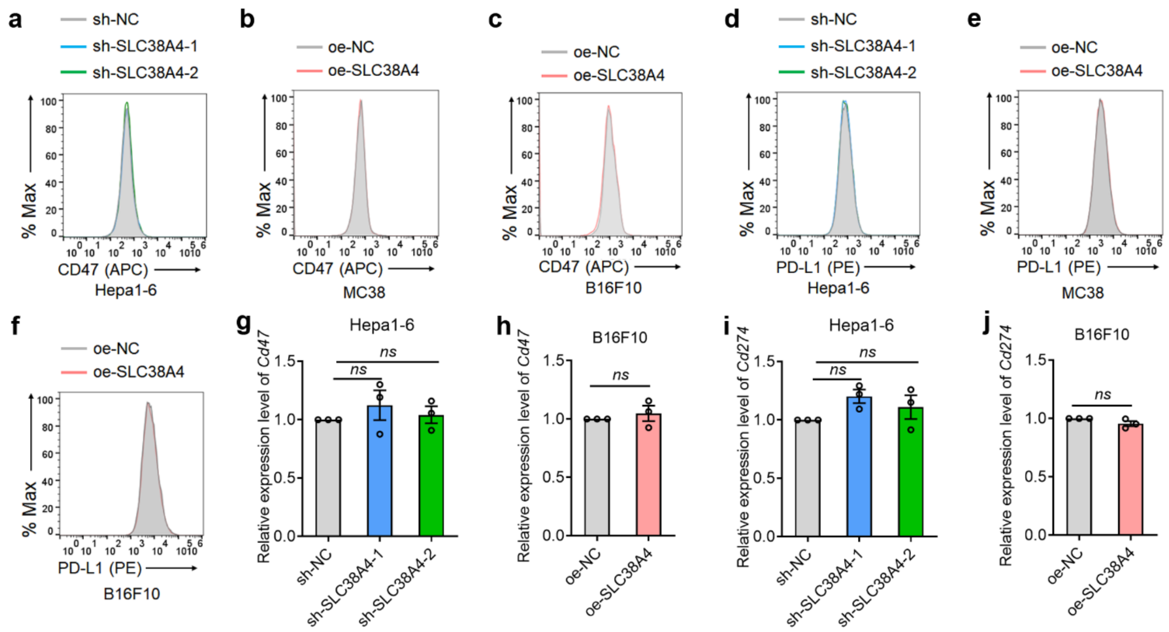

Supplementary Fig. 4 SLC38A4 does not regulate the expression of CD47 and PD-L1. a CD47 protein level in Hepa1-6 cells with SLC38A4 knockdown or control was measured by flow cytometry. **b** CD47 protein level in MC38 cells with SLC38A4 overexpression or control was measured by flow cytometry. **c** CD47 protein level in B16F10 cells with SLC38A4 overexpression or control was measured by flow cytometry. **d** PD-L1 protein level in Hepa1-6 cells with SLC38A4 knockdown or control was measured by flow cytometry. **e** PD-L1 protein level in MC38 cells with SLC38A4 overexpression or control was measured by flow cytometry. **f** PD-L1 protein level in B16F10 cells with SLC38A4 overexpression or control was measured by flow cytometry. **g** *Cd47* mRNA level in Hepa1-6 cells with SLC38A4 knockdown or control was measured by qPCR. **h** *Cd47* mRNA level in B16F10 cells with SLC38A4 overexpression or control was measured by qPCR. **i** *Cd274* mRNA level in Hepa1-6 cells with SLC38A4 knockdown or control was measured by qPCR. **j** *Cd274*

mRNA level in B16F10 cells with SLC38A4 overexpression or control was measured by qPCR. Results are shown as mean ± s.d. of n = 3 independent experiments. ns, not significant, by one-way ANOVA followed by Dunnett's multiple comparisons test (**g**, **i**) or Student’s *t* test (**h**, **j**).

Supplementary Figure 5

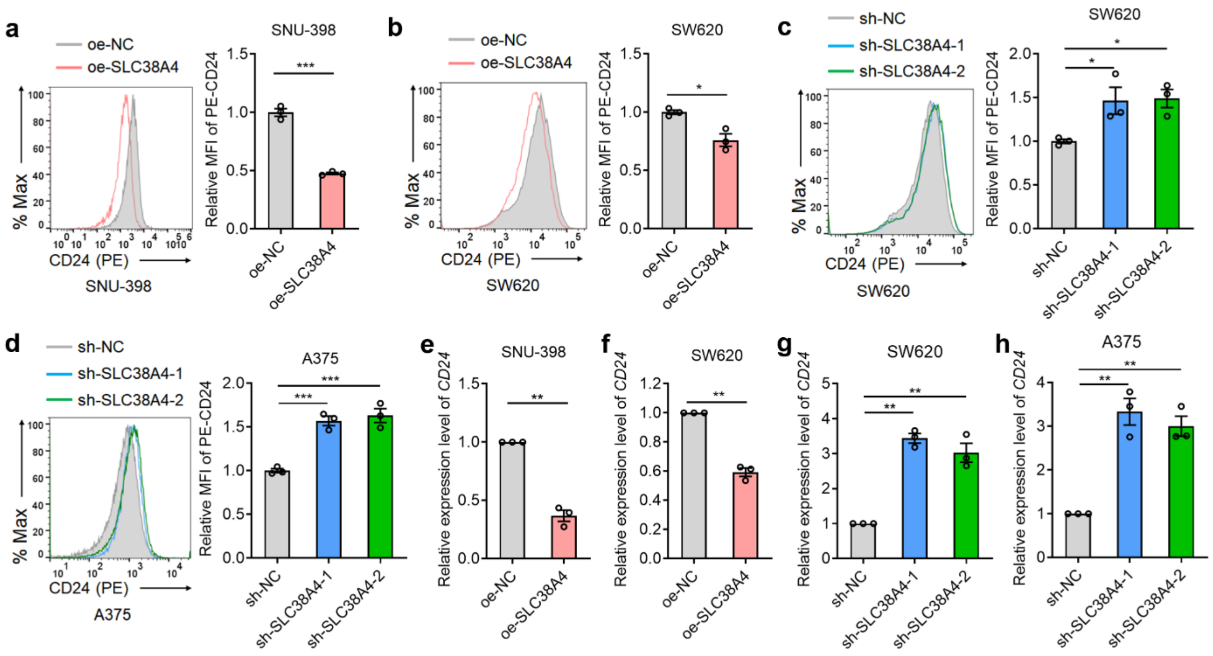

Supplementary Fig. 5 SLC38A4 downregulates CD24 expression in human tumor cells. a CD24 protein level in SNU-398 cells with SLC38A4 overexpression or control was measured by flow cytometry. **b** CD24 protein level in SW620 cells with SLC38A4 overexpression or control was measured by flow cytometry. **c** CD24 protein level in SW620 cells with SLC38A4 knockdown or control was measured by flow cytometry. **d** CD24 protein level in A375 cells with SLC38A4 knockdown or control was measured by flow cytometry. **e** *CD24* mRNA level in SNU-398 cells with

SLC38A4 overexpression or control was measured by qPCR. **f** *CD24* mRNA level in SW620 cells with SLC38A4 overexpression or control was measured by qPCR. **g** *CD24* mRNA level in SW620 cells with SLC38A4 knockdown or control was measured by qPCR. **h** *CD24* mRNA level in A375 cells with SLC38A4 knockdown or control was measured by qPCR. Results are shown as mean ± s.d. of n = 3 independent experiments. **p* < 0.05, ***p* < 0.01, ****p* < 0.001 by Student's *t* test (**a**, **b**, **e**, **f**) or one-way ANOVA followed by Dunnett's multiple comparisons test (**c**, **d**, **g**, **h**).

Supplementary Figure 6

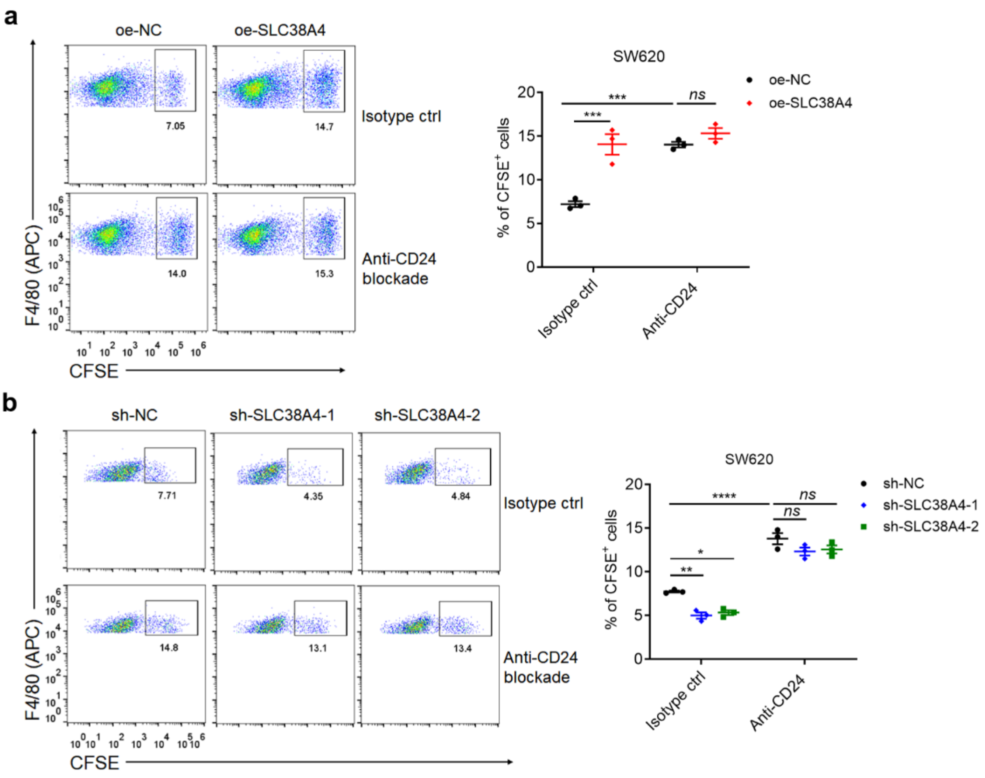

Supplementary Fig. 6 SLC38A4 enhances Kupffer cell-mediated phagocytosis of human tumor cells in a CD24-depedent manner. a In vitro phagocytosis of CFSE-

labeled SW620 cells with SLC38A4 overexpression or control by Kupffer cells in the presence or absence of CD24 neutralizing antibody was measured by flow cytometry. **b** In vitro phagocytosis of CFSE-labeled SW620 cells with SLC38A4 knockdown or control by Kupffer cells in the presence or absence of CD24 neutralizing antibody was measured by flow cytometry. Results are shown as mean ± s.d. of n = 3 independent experiments. *$p < 0.05$, **$p < 0.01$, ***$p < 0.001$, ****$p < 0.0001$, ns, not significant, by two-way ANOVA followed by Tukey's multiple comparisons test.

Supplementary Figure 7

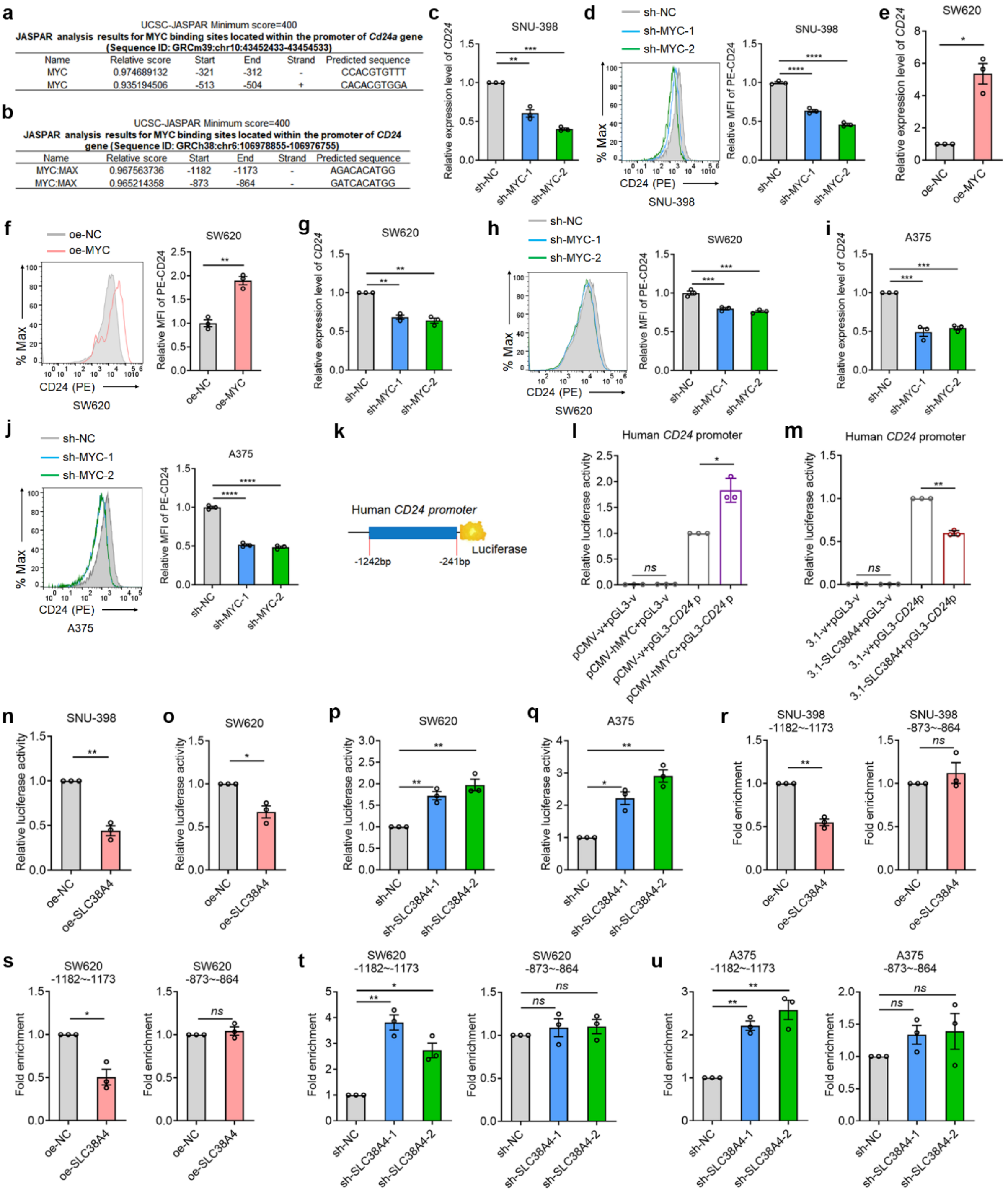

Supplementary Fig. 7 SLC38A4 and MYC suppress *CD24* transcription. a MYC binding sites in mouse *Cd24a* promoters, predicted by JASPAR. **b** MYC binding sites in human *CD24* promoters, predicted by JASPAR. **c** *CD24* mRNA level in SNU-398 cells with MYC knockdown or control was measured by qPCR. **d** CD24 protein level

in SNU-398 cells with MYC knockdown or control was measured by flow cytometry. **e** *CD24* mRNA level in SW620 cells with MYC overexpression or control was measured by qPCR. **f** CD24 protein level in SW620 cells with MYC overexpression or control was measured by flow cytometry. **g** *CD24* mRNA level in SW620 cells with MYC knockdown or control was measured by qPCR. **h** CD24 protein level in SW620 cells with MYC knockdown or control was measured by flow cytometry. **i** *CD24* mRNA level in A375 cells with MYC knockdown or control was measured by qPCR. **j** CD24 protein level in A375 cells with MYC knockdown or control was measured by flow cytometry. **k** Schematic of the luciferase reporter containing human *CD24* promoter. **l** Luciferase activity in 293T cells co-transfected with MYC overexpression vector, luciferase reporter containing *CD24* promoter, and pRL-TK vector which encodes Renilla luciferase. Data are shown as the relative ratio of firefly luciferase activity to renilla luciferase activity. **m** Luciferase activity in 293T cells co-transfected with SLC38A4 overexpression vector, luciferase reporter containing *CD24* promoter, and pRL-TK vector. Data are shown as the relative ratio of firefly luciferase activity to renilla luciferase activity. **n**, **o** Luciferase activity in SNU-398 (**n**) or SW620 (**o**) cells with SLC38A4 overexpression or control, co-transfected with luciferase reporter containing *CD24* promoter and pRL-TK vector. Data are shown as the relative ratio of firefly luciferase activity to renilla luciferase activity. **p**, **q** Luciferase activity in SW620 (**p**) or A375 (**q**) cells with SLC38A4 knockdown or control, co-transfected with luciferase reporter containing *CD24* promoter and pRL-TK vector. Data are shown as the relative ratio of firefly luciferase activity to renilla

luciferase activity. **r**, **s** CUT&RUN assays followed by qPCR were performed in SNU-398 (**r**) or SW620 (**s**) cells with SLC38A4 overexpression or control, to detect the binding of MYC to *CD24* promoter region covering the -1173 site or -864 site. **t**, **u** CUT&RUN assays followed by qPCR were performed in SW620 (**t**) or A375 (**u**) cells with SLC38A4 knockdown or control, to detect the binding of MYC to *CD24* promoter region covering the -1173 site or -864 site. Results are shown as mean ± s.d. of n = 3 independent experiments. **p* < 0.05, ***p* < 0.01, ****p* < 0.001, *****p* < 0.0001, ns, not significant, by one-way ANOVA followed by Dunnett's multiple comparisons test (**c**, **d**, **g-j**, **p**, **q**, **t**, **u**) or Student's *t* test (**e**, **f**, **l-o**, **r**, **s**).

Supplementary Figure 8

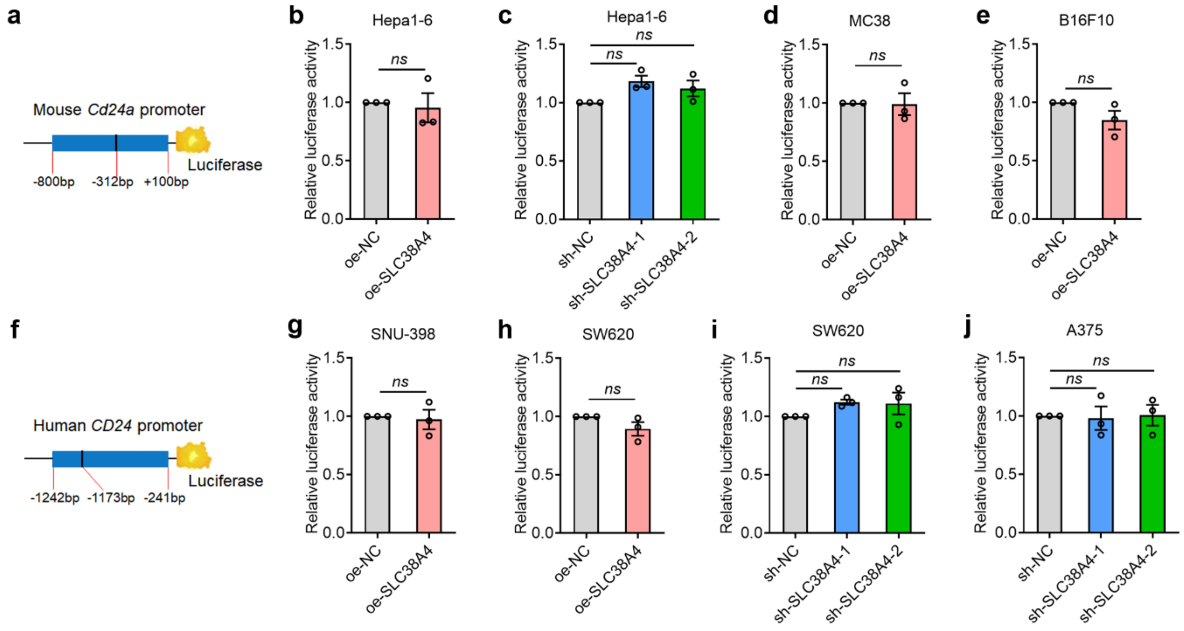

Supplementary Fig. 8 SLC38A4 suppresses *Cd24a*/*CD24* promoter activity through MYC. **a** Schematic of the luciferase reporter containing mutated mouse *Cd24a* promoter. **b**, **c** Luciferase activity in Hepa1-6 cells with SLC38A4

overexpression (**b**) or knockdown (**c**), co-transfected with luciferase reporter containing mutated *Cd24a* promoter and pRL-TK vector. Data are shown as the relative ratio of firefly luciferase activity to renilla luciferase activity. **d**, **e** Luciferase activity in MC38 (**d**) or B16F10 (**e**) cells with SLC38A4 overexpression or control, co-transfected with luciferase reporter containing mutated *Cd24a* promoter and pRL-TK vector. Data are shown as the relative ratio of firefly luciferase activity to renilla luciferase activity. **f** Schematic of the luciferase reporter containing mutated human *CD24* promoter. **g**, **h** Luciferase activity in SNU-398 (**g**) or SW620 (**h**) cells with SLC38A4 overexpression or control, co-transfected with luciferase reporter containing mutated *CD24* promoter and pRL-TK vector. Data are shown as the relative ratio of firefly luciferase activity to renilla luciferase activity. **i**, **j** Luciferase activity in SW620 (**i**) or A375 (**j**) cells with SLC38A4 knockdown or control, co-transfected with luciferase reporter containing mutated *CD24* promoter and pRL-TK vector. Data are shown as the relative ratio of firefly luciferase activity to renilla luciferase activity. Results are shown as mean ± s.d. of n = 3 independent experiments. ns, not significant, by Student's *t* test (**b**, **d**, **e**, **g**, **h**) or one-way ANOVA followed by Dunnett's multiple comparisons test (**c**, **i**, **j**).

Supplementary Figure 9

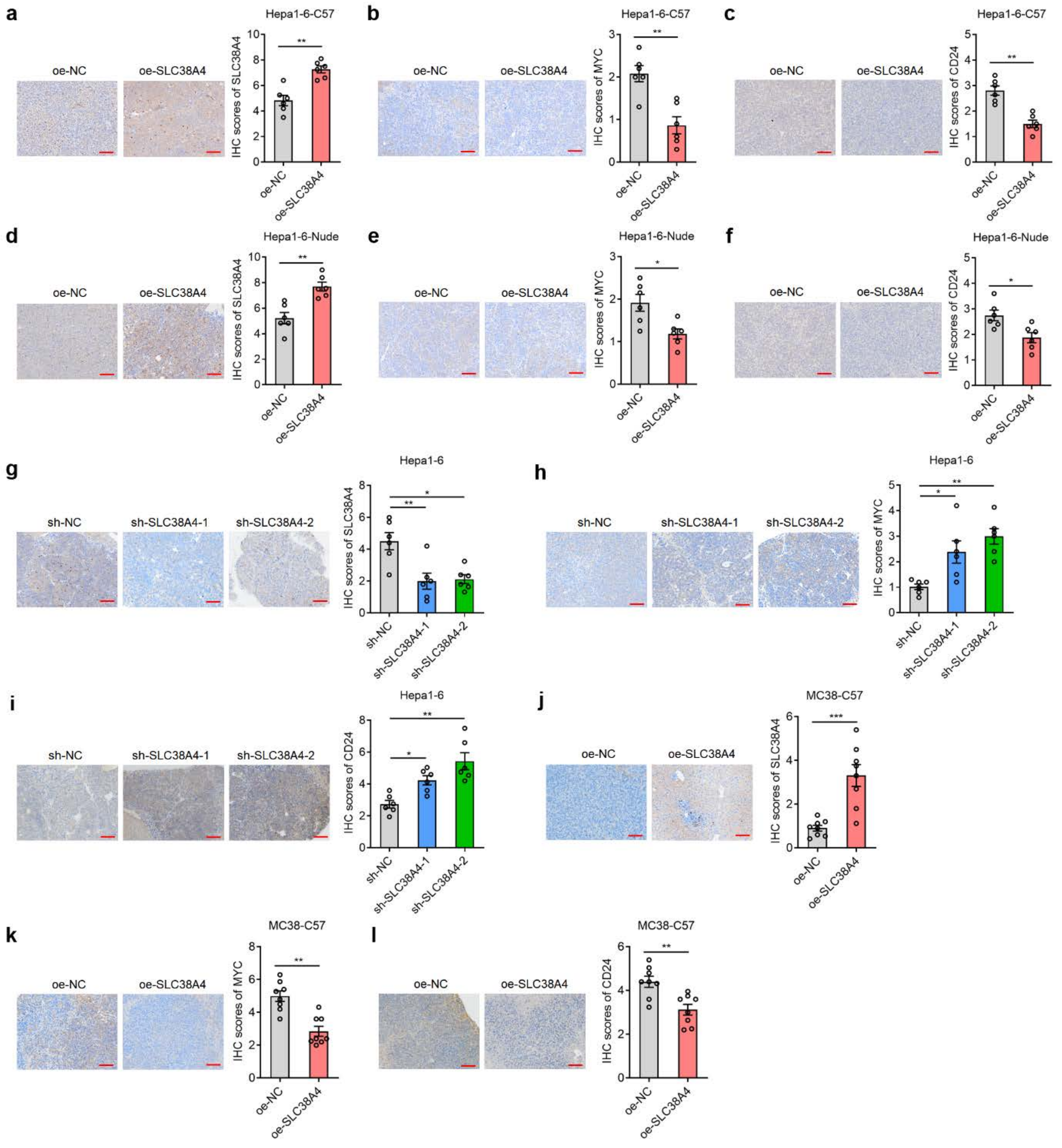

Supplementary Fig. 9 SLC38A4, MYC, and CD24 expression in liver metastatic tissues formed by Hepa1-6 and MC38 cells with SLC38A4 overexpression or knockdown. a-c IHC staining of SLC38A4 (**a**), MYC (**b**), and CD24 (**c**) in liver metastatic tissues formed by SLC38A4-overexpressing and control Hepa1-6 cells in C57BL/6 mice. Scale bars, 100 μm. **d-f** IHC staining of SLC38A4 (**d**), MYC (**e**), and

CD24 (**f**) in liver metastatic tissues formed by SLC38A4-overexpressing and control Hepa1-6 cells in nude mice. Scale bars, 100 µm. **g-i** IHC staining of SLC38A4 (**g**), MYC (**h**), and CD24 (**i**) in liver metastatic tissues formed by SLC38A4-knockdown and control Hepa1-6 cells in C57BL/6 mice. Scale bars, 100 µm. **j-l** IHC staining of SLC38A4 (**j**), MYC (**k**), and CD24 (**l**) in liver metastatic tissues formed by SLC38A4-overexpressing and control MC38 cells in C57BL/6 mice. Scale bars, 100 µm. Results are shown as mean ± s.d. of n = 6 (**a-i**) or n = 8 (**j-l**) mice in each group. *$p < 0.05$, **$p < 0.01$, ***$p < 0.001$ by Mann-Whitney test (**a-f**, **j-l**) or Kruskal-Wallis test followed by Dunn's multiple comparisons test (**g-i**).

Supplementary Figure 10

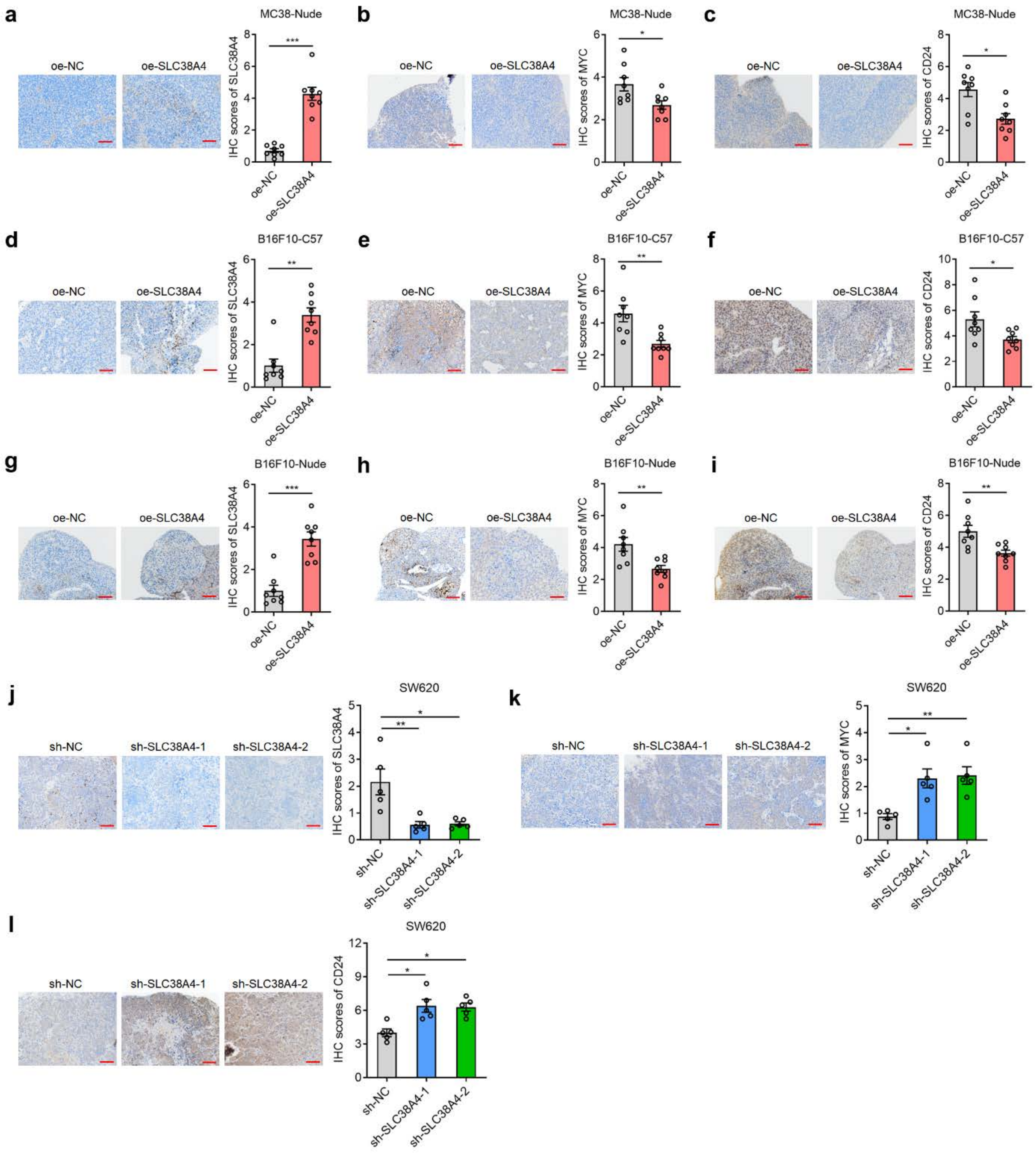

Supplementary Fig. 10 SLC38A4, MYC, and CD24 expression in liver metastatic tissues formed by MC38, B16F10 and SW620 cells with SLC38A4 overexpression or knockdown. a-c IHC staining of SLC38A4 (**a**), MYC (**b**), and CD24 (**c**) in liver metastatic tissues formed by SLC38A4-overexpressing and control MC38 cells in

nude mice. Scale bars, 100 µm. **d-f** IHC staining of SLC38A4 (**d**), MYC (**e**), and CD24 (**f**) in liver metastatic tissues formed by SLC38A4-overexpressing and control B16F10 cells in C57BL/6 mice. Scale bars, 100 µm. **g-i** IHC staining of SLC38A4 (**g**), MYC (**h**), and CD24 (**i**) in liver metastatic tissues formed by SLC38A4-overexpressing and control B16F10 cells in nude mice. Scale bars, 100 µm. **j-l** IHC staining of SLC38A4 (**j**), MYC (**k**), and CD24 (**l**) in liver metastatic tissues formed by SLC38A4-knockdown and control SW620 cells in nude mice. Scale bars, 100 µm. Results are shown as mean ± s.d. of n = 8 (**a-i**) or n = 5 (**j-l**) mice in each group. $^*p < 0.05$, $^{**}p < 0.01$, $^{***}p < 0.001$ by Mann-Whitney test (**a-i**) or Kruskal-Wallis test followed by Dunn's multiple comparisons test (**j-l**).

Supplementary Figure 11

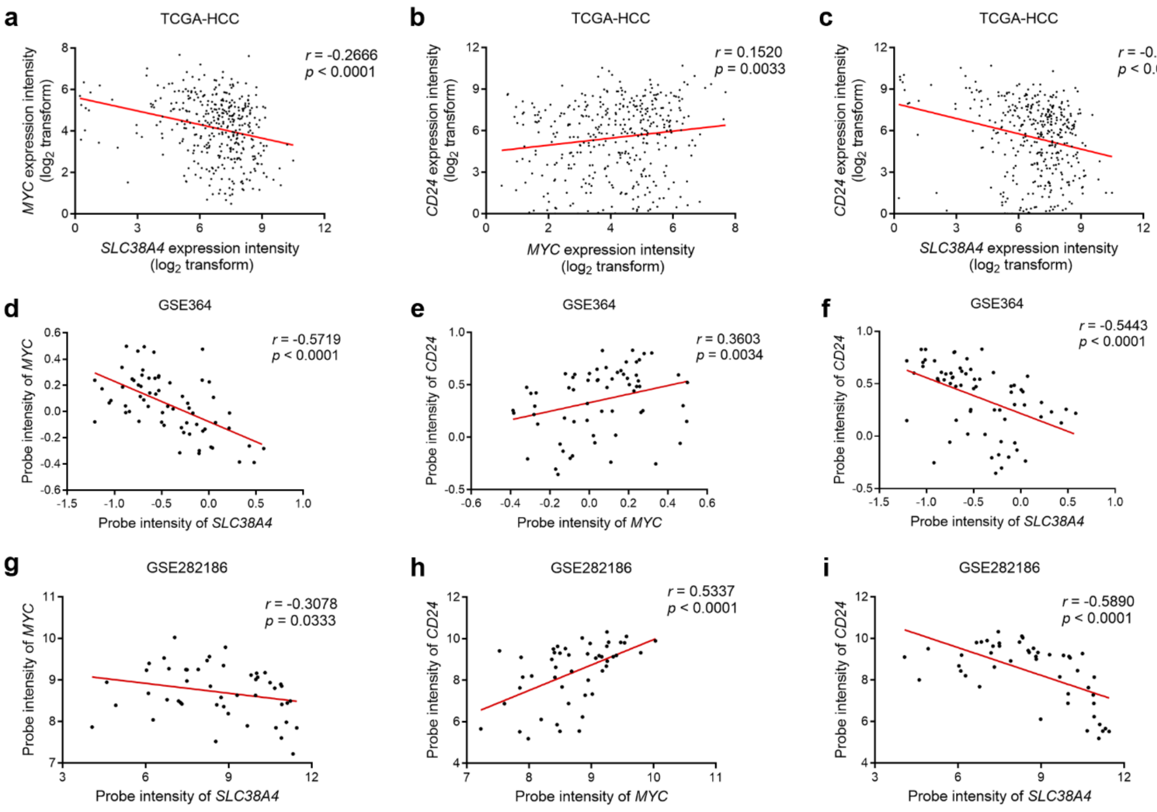

Supplementary Fig. 11 The correlation between SLC38A4, MYC, and CD24 expression in human tissues. a The correlation between SLC38A4 and MYC expression in 371 HCC tissues, based on TCGA data. r = -0.2666, p < 0.0001 by Spearman correlation analysis. **b** The correlation between MYC and CD24 expression in 371 HCC tissues, based on TCGA data. r = 0.1520, p = 0.0033 by Spearman correlation analysis. **c** The correlation between SLC38A4 and CD24 expression in 371 HCC tissues, based on TCGA data. r = -0.2381, p < 0.0001 by Spearman correlation analysis. **d** The correlation between SLC38A4 and MYC expression in 67 metastatic HCC tissues, based on GSE364 data. r = -0.5719, p < 0.0001 by Spearman correlation analysis. **e** The correlation between MYC and CD24 expression in 67 metastatic HCC tissues, based on GSE364 data. r = 0.3603, p = 0.0034 by Spearman

correlation analysis. **f** The correlation between SLC38A4 and CD24 expression in 67 metastatic HCC tissues, based on GSE364 data. $r = -0.5443$, $p < 0.0001$ by Spearman correlation analysis. **g** The correlation between SLC38A4 and MYC expression in 48 CRC liver metastatic tissues, based on GSE282186 data. $r = -0.3078$, $p = 0.0333$ by Spearman correlation analysis. **h** The correlation between MYC and CD24 expression in 48 CRC liver metastatic tissues, based on GSE282186 data. $r = 0.5337$, $p < 0.0001$ by Spearman correlation analysis. **i** The correlation between SLC38A4 and CD24 expression in 48 CRC liver metastatic tissues, based on GSE282186 data. $r = -0.5890$, $p < 0.0001$ by Spearman correlation analysis.
