# Supplementary figures and images for: SLC38A4 promotes Kupffer cell phagocytosis and suppresses tumor liver metastasis
Source: Exp Mol Med. 2026 May 1;58(5):1425–38. doi: 10.1038/s12276-026-01703-5 (PMC13234026; doi:10.1038/s12276-026-01703-5)

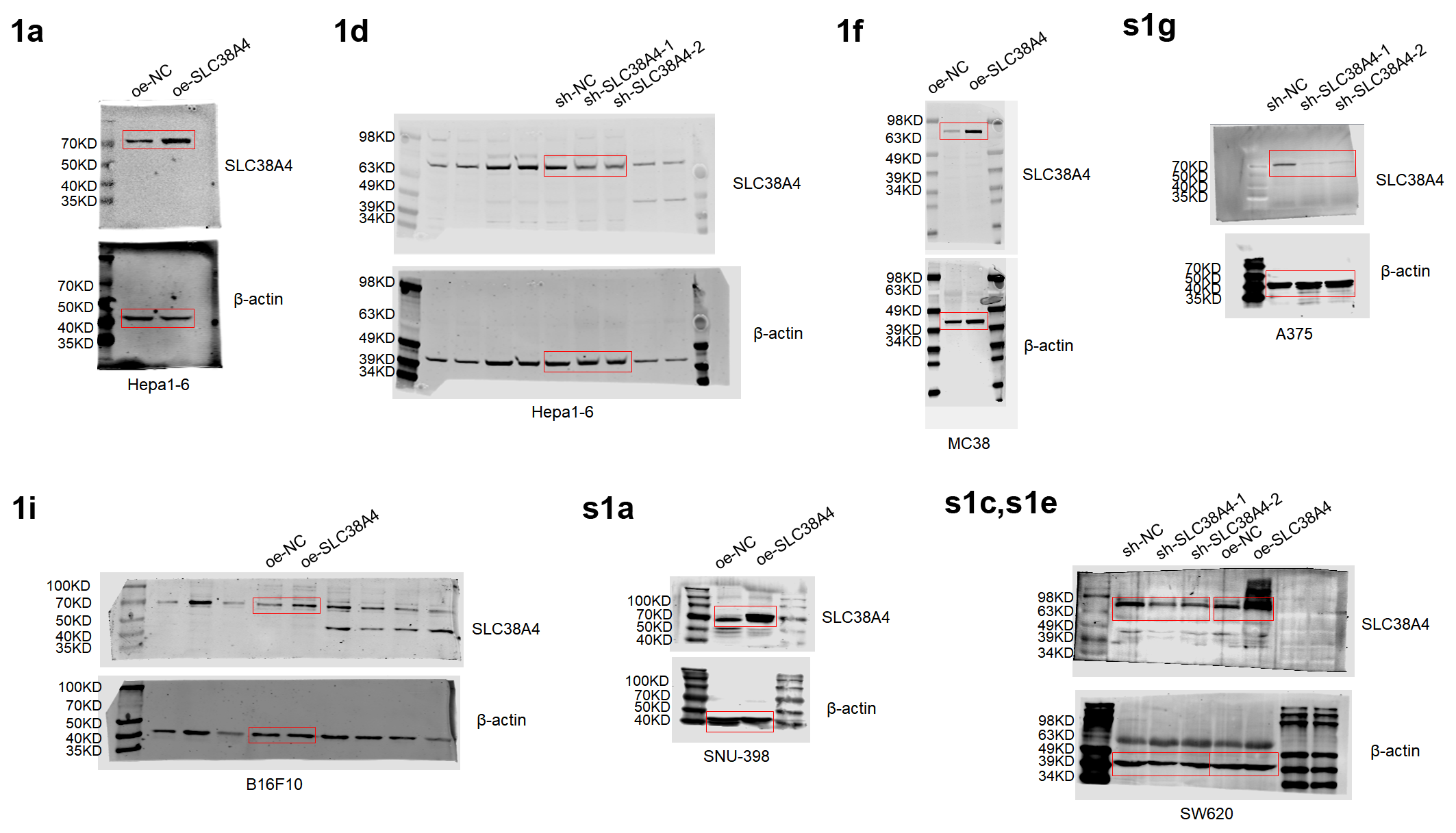

Supplement: Supplementary file 2 — raw western blots [file 12276_2026_1703_MOESM2_ESM.tif]
